# An RCT study showing few weeks of music lessons enhance audio-visual temporal processing

**DOI:** 10.1038/s41598-022-23340-4

**Published:** 2022-11-22

**Authors:** Yuqing Che, Crescent Jicol, Chris Ashwin, Karin Petrini

**Affiliations:** 1 Department of Psychology, University of Bath, Claverton Down, Bath, BA2 7AY UK; 2 Department of Computer Science, University of Bath, Claverton Down, Bath, BA2 7AY UK; 3 Centre for Applied Autism Research, Department of Psychology, University of Bath, , Claverton Down, Bath, BA2 7AY, UK, Bath, UK

**Keywords:** Psychology, Human behaviour

## Abstract

Music involves different senses and is emotional in nature, and musicians show enhanced detection of audio-visual temporal discrepancies and emotion recognition compared to non-musicians. However, whether musical training produces these enhanced abilities or if they are innate within musicians remains unclear. Thirty-one adult participants were randomly assigned to a music training, music listening, or control group who all completed a one-hour session per week for 11 weeks. The music training group received piano training, the music listening group listened to the same music, and the control group did their homework. Measures of audio-visual temporal discrepancy, facial expression recognition, autistic traits, depression, anxiety, stress and mood were completed and compared from the beginning to end of training. ANOVA results revealed that only the music training group showed a significant improvement in detection of audio-visual temporal discrepancies compared to the other groups for both stimuli (flash-beep and face-voice). However, music training did not improve emotion recognition from facial expressions compared to the control group, while it did reduce the levels of depression, stress and anxiety compared to baseline. This RCT study provides the first evidence of a causal effect of music training on improved audio-visual perception that goes beyond the music domain.

## Introduction

Music brings joy and consolation to humans which greatly enriches people’s lives. However, playing musical instruments is demanding and engages several sensory and motor systems and many high-level cognitive processes^1–3^. When playing a musical instrument, the musician needs to read the music scores, generate movement accordingly, and monitor the auditory and tactile feedback to adjust further actions^4^. For example, learning to play the piano requires intensive coupling of the visual cues (reading the music scores and monitoring the finger movements) with the auditory cues (the sounds that the piano makes), which results in a multisensory training that can benefit audio-visual processing and enhance the ability to use this information. Moreover, music is an art with an expressive nature. Musicians need to perceive and interpret the emotional undertone of a musical piece and then manipulate the acoustic characteristics such as tempo, dynamics and valences to convey the intended feelings and emotions to the audience^5,6^. Indeed, Olszewska et al.^7^ proposed that music training provides a framework for studying the brain and behavioural plasticity because it introduces a novel activity with complex sensorimotor behaviour, adaptations related to learned behaviours, and involvement of higher-order cognitive functions. Given the multisensory and emotional nature of music practice, it is not surprising that musicians are reported to have better multisensory^8,9^ and emotion processing abilities^10,11^ than non-musicians. As multisensory and emotion processes are both crucial for daily cognitive functions and social interactions, a deeper understanding of how music could enhance them will inform the development of cognitive training for people who are known to have difficulties in multisensory^12,13^ and emotion processing^14^.

A growing body of studies suggests that musicians have an enhanced ability to detect asynchrony between visual and auditory cues, which is demonstrated by a narrower audio-visual synchrony window (ASW), i.e., a smaller range of asynchrony perceived as synchronous^8,9,15^. Lee and Noppeney^9,16^ and Petrini et al.^8,17^ found that pianists and drummers have significantly narrower ASW for audio-visual piano and drumming clips respectively. They concluded that the behavioural enhancement is reflected in changes in activity and connectivity in multisensory and time-keeping areas such as the cerebellum and the superior temporal sulcus. This fine-tuning effect of long-term music training on audio-visual perception has since been replicated with further non-musical stimuli such as flash and beep^15,18,19^. However, a reduced ASW for musicians has not been reported by all studies and for all types of stimuli^16,20^. Lee and Noppeney^9^ found that musicians’ ASW for audio-visual speech clips was comparable to that of non-musicians, suggesting that the effect of musicianship on audio-visual synchrony perception may not transfer to domains beyond music.

Besides being a natural multisensory activity, music is also an important means of expression and communication^21^. Facial and body expressions are essential to musical performance and are used by musicians to communicate between co-performers and audiences^22^. Consequently, musical expertise has been found to have an enhanced effect on emotion processing for musical stimuli^23–25^. The current consensus is that musicians can better perceive and categorise musical emotions than non-musicians^26^. For example, Lima and Castro^10^ asked musically trained and untrained participants to rate the expressed emotion (happiness, sadness, peacefulness, and fear) in a set of musical excerpts. They found that musicians were more accurate in emotion recognition for all four emotions. The enhanced ability of musicians to recognise emotions also extends to speech prosody. For example, studies have found a positive correlation between music training length and accuracy of emotion recognition from speech prosody^10^. This is not surprising given that music and speech share many key acoustic features such as tempo, pitch, loudness, and timbre^27–30^, and because there is a universal association between certain acoustic cues and emotions^31^. Whether musicians have an enhanced ability to recognise emotions from information other than sound, such as facial expressions, is currently unclear. For example, Correia et al.^32^ found that musicians did not outperform non-musicians in a facial expression recognition task.

Music is also well-known for its benefits on mood and wellbeing in both typically developed^33,34^ and autistic people^35,36^ as it can reduce stress and depression. However, most of these studies have focused on music listening rather than music training^33,34^. The fundamental differences in the sensory and emotional engagement between these two activities raises the question about whether music practice could also benefit mood and wellbeing. For example, Seinfeld et al.^37^ showed that a four-month piano training period could significantly decrease depression and induce positive mood states in older adults. However, whether this positive effect of music training extends to a younger population remains unclear.

The evidence pointing to a positive effect of music training on cognitive and emotional mechanisms comes from cross-sectional studies comparing musicians and non-musicians. Stronger evidence for this effect would be provided by randomised controlled design (RCT) studies. However, RCT trials testing the effects of music training on cognitive and emotional enhancements have been scarce to date. Preliminary evidence on the effect of music training on audio-visual synchrony perception comes from the study by Gerson et al.^38^ study carried out with infants. They used a preferential-looking paradigm and studied the effect of drumming practice on audio-visual temporal processing. They found that infants who received a five-minutes drumming session before the task, compared to those who did not, had significantly longer looking time for the synchronous drumming video display than the asynchronous video. Gerson et al.^38^ demonstrated that even a short period of active music experience could affect audio-visual synchrony perception. Two RCT studies have shown the enhancing effect of music training on emotion recognition from speech prosody^39,40^. For example, Thompson et al.^39^ found that six-year-olds who received one year of regular keyboard training showed greater emotion recognition from speech prosody compared to age-matched controls who received singing training, although they did not outperform those children who took drama classes). Similarly, Good et al.^40^ found that children with cochlear implants’ improved in a speech prosody task after a 24-month piano training programme (with a 30 min session per week). To date, no RCT studies have been reported assessing the effect of a short period of music training on adults’ audio-visual synchrony perception and emotion recognition beyond the auditory domain.

Understanding whether music training can improve audio-visual temporal and emotion processing is especially important as these abilities have been shown to be significantly reduced in people with autism spectrum disorders (ASD)^41–45^. Several recent studies have used autistic traits as a proxy to examine variations in these cognitive abilities related to the degree of autistic traits, based on the idea that the level of autistic traits is measurable and evident across the wider non-clinical population^46–48^. These studies have found evidence of altered audio-visual temporal processing and impaired emotion processing in neurotypical individuals with high levels of autistic traits^49–51^. For example, individuals with high levels of autistic traits were found to have wider ASW than individuals with lower levels of autistic traits in communication, speech, and attention switching processing^52,53^, in line with findings from studies involving people with ASD^42,43^. Furthermore, people with high levels of autistic traits, similarly to people with ASD^44^, require higher emotion intensity to recognise emotions from facial expressions^51^. However, whether music training could be effective in enhancing these abilities for individuals with high levels of autistic traits remains unknown.

The current study involved an RCT study to examine whether one hour of piano training per week over eleven weeks could enhance audio-visual synchrony perception (reduce the size of the ASW) and facial expression recognition. We hypothesed that an eleven-week piano training period would lead to a significant improvement in audio-visual temporal processing, i.e., a narrower ASW in neurotypical adults for simple stimulus (e.g., flash-beep). We did not have a clearer prediction for the effect of music training on complex audio-visual stimuli (e.g., face-voice) or emotion recognition from facial expressions, as the evidence from previous research is inconclusive or insufficient. We also asked whether such improvement would be more evident for people with high levels of autistic traits and whether music lessons could have a positive effect on mood and depression, anxiety and stress, given that music listening has been shown to have positive effects on depression, anxiety and stress^33,34,54–57^ as well as mood^58,59^ while less clear results are currently available for music training^60^.

Hence, this study is novel in many respects. It is the first RCT study to examine the effect of music training on ASW in adults using non-music audio-visual stimuli of different complexity, thus testing whether music is the key to enhancing this ability (beyond the music and sound domain) rather than other predeterminants. It is also the first RCT study examining the effect of music training on emotion recognition using visual stimuli, thus extending investigations beyond the sound and music domain to better understand the transferability of music training effects. It is the first RCT study examining the effect of music training compared to music listening on mood and depression, anxiety and stress. Finally, given that cross-sectional studies have compared musicians with several years of training to non-musicians, it remains unclear whether a few weeks of training can be effective in enhancing audio-visual temporal processes as well as emotion recognition abilities, especially when compared to music listening.

## Method

### Design

The current study used a parallel-group RCT design. We did not include blinding in this study as the design required participants’ active involvement in certain conditions, with the experimenter (YC) having to know and run the sessions and to musically train participants. The experimenter had no control over the group allocation process as the participants were randomly assigned to their group at the beginning of the study. Therefore, we compared the performance between the three groups in the first trial to test for any possible experimenter bias effect on the groups’ performance and found no significant difference among groups, suggesting an absence of such bias (see Results section for details about this analysis). Participants were all non-musicians and were randomly assigned to three experimental conditions: a music training group (MT), a music listening group (ML), or a control (C) group that received no musical input. We included the ML group in the current study to control for the music listening factor in the design, as participants in the ML group listened to the same music that was taught in the MT group, any behavioural difference between these two groups by the end of the study could only be explained by the training per se. The first eleven weeks involved training sessions and included seven bi-weekly data-collection sessions (i.e., in week 1, 3, 5, 7, 9, 11 and 13). In a previous perceptual training study for audio-visual temporal processing, Powers et al.^61^, tested participants immediately after the one-hour training session for five successive days with a follow-up test one week after the training. We adapted the testing schedule in Powers et al. to bi-weekly data-collection sessions in the current study to achieve six testing sessions and one follow-up session after two weeks from the end of the study. So while data were collected before the session in week 1 and after the session in week 3, 5, 7, 9, and 11, the music lessons were provided every week for one hour for 11 weeks (i.e., during a three-months period). Data collection was also done at week 13 to measure participants’ performance at a two-week follow-up after the training, such that the entire study lasted thirteen weeks. As mentioned, the experimenter also acted as the music trainer and was present for all the sessions for all participants, similar to the methods in Gerson et al.^38^. All the test sessions were completed in a psychology research laboratory at the University of Bath under quiet conditions.

### Participants

A total sample size of 42 (14 per group) was calculated for a partial equal to 0.06 (for medium effect size) through a priori type of power analysis for an ANOVA repeated measures within-between interaction. We used G*Power 3.1^62^ and assumed a level of power of 0.80, 3 groups, 2 measurements, and an alpha level of 0.05. Participants were initially recruited through university advertisement and were students or staff of the University of Bath and residents of Bath with a similar level of education and socioeconomic status. Participants were randomly assigned to the MT group, ML group, or C group. A total of five participants dropped out early in the study, two in the MT group, two in the ML group, and one in the C group, and we had to stop recruitment and testing early due to COVID-19 restrictions. Hence, the final sample was composed of 31 participants. The participants were from five countries (14 British, 11 Chinese, four Indian, one Malaysian, and one Danish) and were all fluent in English. There were 10 participants in the MT group (*Mean age* = 31.37 years, *SD* = 11.05, six females), 11 participants in the ML group (*Mean age* = 28.03 years, *SD* = 11.35, eight females), and 10 participants in the control group (*Mean age* = 27.89 years, *SD* = 11.02, seven females). The sample size of each group in the current study was similar to, and at times, greated than the previous RCT studies examing the effect of music lessons on perceptual and cognitive abilities^39,40,63^. The mean Autism-Spectrum Quotient (AQ) score, measured by the 50 item AQ^46^, for the entire sample was 18.4 (*SD* = 6.6), which is similar to the mean AQ scores reported for previous large non-clinical samples^64^. All participants self-reported to have normal or corrected-to-normal vision and hearing, no history of neurological disorders or a diagnosis of ASD. They were all right-handed apart from one person in the control group who was born left-handed but changed to using the right hand.

We explicitly recruited people with no music training history besides the compulsory music classes in schools and further screened the participants by asking them to fill in a basic information questionnaire (see supplementary material), which asked them to confirm that they did not have any musical instrument training in the past. Participants with any training history were all thanked for their interest and debriefed about the aim of the study but told that, unfortunately, they could not participate to the study given the exclusion criteria. The basic questionnaire also recorded participants’ age, gender, dominant hand, and average daily music listening hours. As evidence suggests that some musical abilities can be obtained through abundant music listening^65^, we did not recruit participants who reported to listening to music for more than six hours per day. All participants were asked not to take any music training or have any music practice outside the piano lessons for the duration of the study. All participants accepted the same condition before taking part and confirmed at the later sessions that indeed they did not practice music outside the training sessions organised for the study.

### Procedure

Before taking part, all participants gave their written and informed consent. The study received ethical approval from the Psychology Research Ethics Committee of the University of Bath (17–283), and the experiments were conducted in accordance with the institutional guidelines and regulations. The participants in the MT group received 11 free piano lessons, and participants in the ML and C groups received £52.50 for their participation. During the training session, MT participants received an hour-long piano lesson, and the ML group listened to one hour of piano songs taken from the playlist the MT was learning. Participants in the control group read and studied quietly for an hour. The baseline ASW and emotion recognition measures of participants were obtained at the outset of week 1, while the other six data-collection sessions all took place after the music training sessions. Participants completed paper questionnaires and computer-based cognitive tasks independently during each data-collection session. The experimenter strictly followed the same protocol and gave the same instructions script to all participants during the data collection. For example, before the emotion recognition task, the experimenter set up the task on the computer and then instructed the participants to read the instructions on the screen carefully before entering the practice and the test trials. Each data-collection session lasted approximately 30 to 45 min. See supplemental material Table S1–S3 for the detailed training and testing timetables.

### Questionnaires

#### Autism-spectrum quotient 50 items (AQ-50)

The AQ is a self-report questionnaire with 50 items that measure autistic traits in five different domains: social skill, attention to detail, attention switching, communication, and imagination. Participants total score ranges from 0 to 50, with a higher score indicating a higher level of autistic traits. The AQ-50 was completed by participants at the beginning and end of the study (i.e., in week 1 and week 13). The mean score of the two AQ measures was used as the final AQ score. Please see supplemental materials Table S4 for detailed results and analysis.

#### Positive and negative affection schedule (PANAS)

The PANAS^66^ consists of ten positive emotion words (e.g. Proud, Strong) and ten negative words (e.g. Upset, Jittery). Participants rate each emotion word on a five-point Likert scale according to how they are feeling at the moment, ranging from 1 (not at all) to 5 (extremely). The ratings for the positive and negative affect are summed to produce two final scores. This measure was included to investigate the training effect on the participant’s positive and negative mood.

#### Depression anxiety stress scale-21 items (DASS-21)

The DASS-21 is a self-report questionnaire designed to measure the depression, anxiety and stress levels of the participants^67^. Participants score each item by rating their emotional state in the past week from 0 (did not apply to me at all) to 3 (very much applied to me). The scores range from 0 to 63 with a higher score indicating a more negative emotional state in the past week.

### Tasks and stimuli

#### Audio-visual simultaneity judgement task

**Figure 1:**
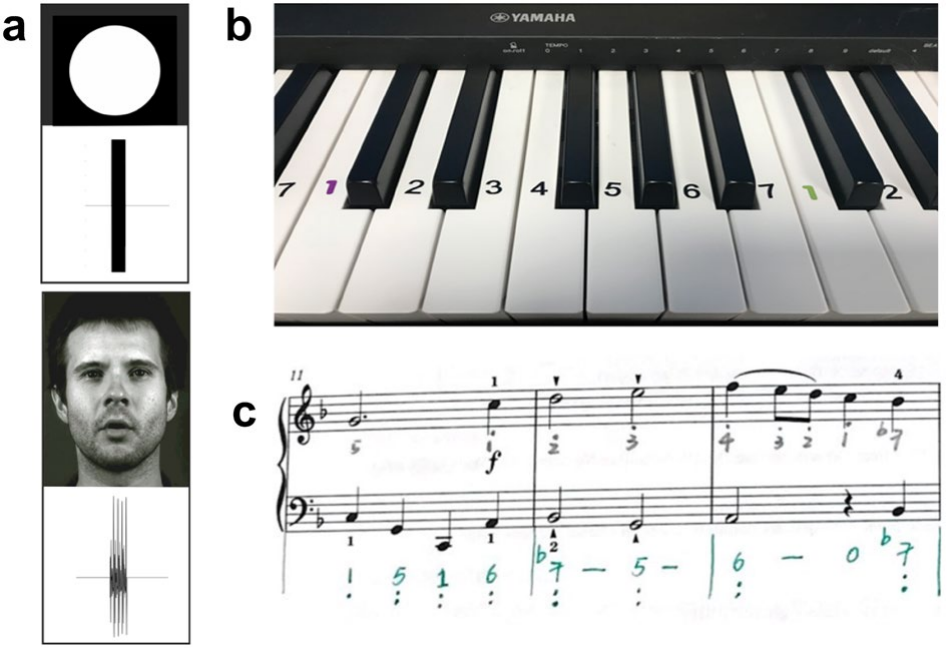
Stimuli and materials illustrations. (**a**) Video frame and waveform representations of the stimuli used in the simultaneity judgement task. The flash-beep (top) consists of a single flash on a black background accompanied by a single beep. The face-voice (bottom) consisted of a male face on a black background accompanied by a ‘o’ utterance. The area size of the white flash approximated that of the mouth area in the face-voice clip. (**b**) Numbered stickers were attached to the keyboard to guide the participants to find the correct key. (**c**) Numbered music notation were presented with the music score, the grey-colour numbers represented the right-hand part and the green-colour numbers represented the left-hand part.

Participants were asked to judge the simultaneity of two types of stimuli, a flash-beep and a face-voice audio-visual clip. The flash-beep stimuli have been used and standardized in previous studies^19^. It consists of a 2000 Hz pure tone with a mean intensity of 84 dB and a rapidly presented white circle (luminance: 85 cd/ m2) in the centre of a black background (luminance: 0.12 cd/ m2). Both the flash and beep were 460 ms in length. The face-voice stimuli were modified from the ‘face-voice’ condition in Love et al.^68^ and^69^, which originally included a dynamic audio-visual clip (25 Hz) with a native English male saying the word ‘‘tomorrow’’. The face-voice clips were presented in black and white, similarly to the flash-beep clips. To obtain face-voice clips resembling the duration (460 ms) and dynamics of the flash-beep clips, we extracted a smaller part of the clip in which the person was pronouncing the second ‘o’ from ‘tomorrow’ (Fig. 1a, the human identity revealing image has been openly published in Love et al.^68^, also see supplemental material for example videos).

During a test session, participants completed a 15-min simultaneity judgement task, where participants had to judge whether the audio and video of the clips were in synch or not. The task was composed of two blocks, one for the flash-beep stimuli and one for the face-voice stimuli. The order of the blocks was counterbalanced across participants. The stimulus onset asynchronies (SOAs) between the auditory and visual information for both stimuli were set at nine levels based on previous studies (−266.67, −200, −133.33, −66.67, 0, 66.67, 133.33, 200, and 266.67 ms; Love et al.^68^. The positive SOAs indicate that the visual cue preceded the auditory cue, and vice versa for the negative SOAs. The audio-visual clip at each level of SOAs was repeated ten times randomly for both flash-beep and face-voice stimuli. Hence, there was a total of 180 trials (9 SOAs × 10 repetitions × 2 blocks) for the task. Before starting the study, participants were given both verbal and written instructions and were given a chance to ask questions or clarification to the experimenter. A practice trial in Week 1 for both stimuli (9 SOAs $\times$ 2 stimuli) was included to familiarize participants with the task. Participants pressed the space key to start the test. Each trial began with a one-second prompt with ‘TEST CLIP’ written in white font presented on a black background. After the prompt, an audio-visual clip was presented, followed by instructions for participants to make a judgement. They were asked to press ‘1’ if they perceived the audio-visual cues to be in-synch, and press ‘3’ if not. Immediately after they submitted their response, the next trial started.

All stimuli were presented via a 13-inch Apple MacBook Pro with Retina display (60 Hz refresh rate) laptop running OS 10.13.6 and an Intel Iris Plus Graphics 650 1536 MB graphics card with 8 GB of LPDDR3 memory. The visual cues were displayed by the retina screen in full screen (resolution: 2560 × 1600). Participants were seated approximately 45 cm from the screen. The auditory cues were presented with the built-in speaker. The volume was set at 60 dB intensity for both the flash-beep and the face-voice. The SJ task program was controlled using MATLAB 2017b (MATHWORKS Inc., Natick, MA) and the Psych-Toolbox^70^.

#### Emotion recognition task (ER)

Facial expression videos from the Amsterdam Dynamic Facial Expression Set -Bath Intensity variations (ADFES-BIV)^71^ were used as stimuli for the emotion recognition task. The ADFES-BIV originally had 12 encoders (seven males) portraying 10 emotions at three intensities (low, intermediate, and high). As no difference was found between the encoders’ expressiveness in the study^71^, the present study only included the videos of six encoders (three males) from the original set expressing the six basic emotions (happiness, sadness, fear, anger, surprise and disgust) and neutral. This produced a total of 126 (7 emotions * 3 intensities * 6 encoders) video clips used in the current study which were randomly presented to participants across 4 separate blocks. All clips were 1040 ms in length. They started with an encoder showing a neutral expression and then developed into one of the seven emotions.

The apparatus and settings were the same as the simultaneity judgement task. Before starting the study, participants received verbal and written instructions and were given a chance to ask any questions to the experimenter. Participants then pressed the space key to continue. A practice trial for all emotions was included to familiarize them with the task. A different encoder was presented in these clips, and these seven clips were not included in the experiment. All participants completed the practice trials at week 1. During the test, a 500 ms fixation cross was first shown on the screen, followed by an emotion clip. A 500 ms blank screen was presented after the clip which was followed by the emotion categories being listed on the screen. Participants were instructed to drag the mouse on the screen using the MacBook touchpad and select one of the emotion categories as fast as possible. The next trial started immediately after a response was recorded. The mouse appeared at a random position on the screen during the testing phase to ensure an even distance from each emotion category. Participants’ reaction times and accuracy were recorded.

### The music training

Participants in the MT group received one-to-one piano lessons in a soundproof room in the Cross-modal laboratory at the University of Bath. The lessons were given by the experimenter, who had 12 years of private piano education in China and obtained a certificate equivalent to ABRSM piano grade 8. A Yamaha digital piano P-35 was used for the lessons. The positions of the participant and the experimenter and the light and volumes within the room were all kept constant throughout.

Before the first lesson, participants received an extra 10-min of tuition on basic musical concepts such as the note duration, the staff, clefs, time signature, steps, and accidentals. They were also taught the appropriate hand and finger postures for piano playing. As no one had any music training before, they were unable to read the musical score. To address this, the music sheets were transcribed into numbered musical notation. Self-adhesive number stickers were attached to each key to provide further guidance (Fig. 1b,c).

We adapted the training content from the classic training regime widely used in China and many other countries (finger practice + songs). Each lesson included two segments. The first 20-min segment was dedicated to finger exercises using the Hanon: the Virtuoso Pianist, Book 1: in Sixty Exercises for the Piano (ISBN: 978-0-7935-5121-7)^72^, which is a progressive practice widely used for beginners and it is excellent for building finger strength, independence, and flexibility. The notes played by both hands are exactly the same, and the finger pattern for each music measure is repetitive^73^. Minimal skills and music knowledge are required for the Hanon. It is intense yet also fast to begin with, making it ideal for effective music training in this study. The second segment consisted of learning a song from the ABRSM 2017–2018 piano grade one exam list for 40 min^74^. The ABRSM is an education body and the exam board of the Royal Schools of Music, and it issues a standardised exam piece every year. The piano grades range from one to eight, with grade one being the elementary level. Five songs were chosen from the list (see example Fig. S1 and detailed training/listening curriculum in the supplemental material). Participants started with the first song ‘A Stately Sarabande’. They proceeded to the next song once they had learned the previous one. Participants in the MT group attended a one-hour weekly lesson for 11 weeks. All participants were asked not to engage in any music training and practice during the three-month study period.

### Data estimation and exclusion for ASW

The number of synchrony responses as a function of SOAs for each participant were fitted with a normal Gaussian function. This method has been widely adopted by previous researchers^17,75^. The size of ASW was then derived from the fit^19,68^. An individual measure of ASW was calculated for both stimuli (flash-beep and face-voice) for each testing session (i.e., in week 1, 3, 5, 7, 9, 11 and 13), which results in 14 ASW measures for each participant by the end of the study. The last testing session for the study was at week 11 as the testing session at week 13 reflected a two-week follow-up measurement after the end of the study. The fitting of the Gaussian function to the data also returned measures of ${R}^{2}$ which represent how well the function fitted the data. In line with previous studies^68,76^ we excluded values of ${R}^{2}$ with a score below or equal to 0.5 which indicate that participants were unable to do the task, and values of ASW that exceeded the range of SOAs used in the current study. Using these criteria, two ASW values for one participant had to be excluded in the MT for the flash-beep condition, while none had to be excluded for the face-voice condition. In the ML one ASW value for four participants had to be excluded in the flash-beep condition, while only one ASW value for one participant in the face-voice condition was excluded (see supplemental materials Fig. S2). Finally, for the control group we excluded two ASW values for two participants, and three ASW values for one participant in the flash-beep condition, while one value of ASW for two participants in the face-voice condition was excluded. For participants where the excluded ASW value coincided with the first (week 1) or last testing session (week 11), then the change in ASW was calculated using the first and last ASW value for that participant. This was the case for two participants (one in the MT and one in the ML) who could not attend the last one or two testing trials due to COVID-19 restrictions.

## Results

### Audio-visual simultaneity perception

**Figure 2:**
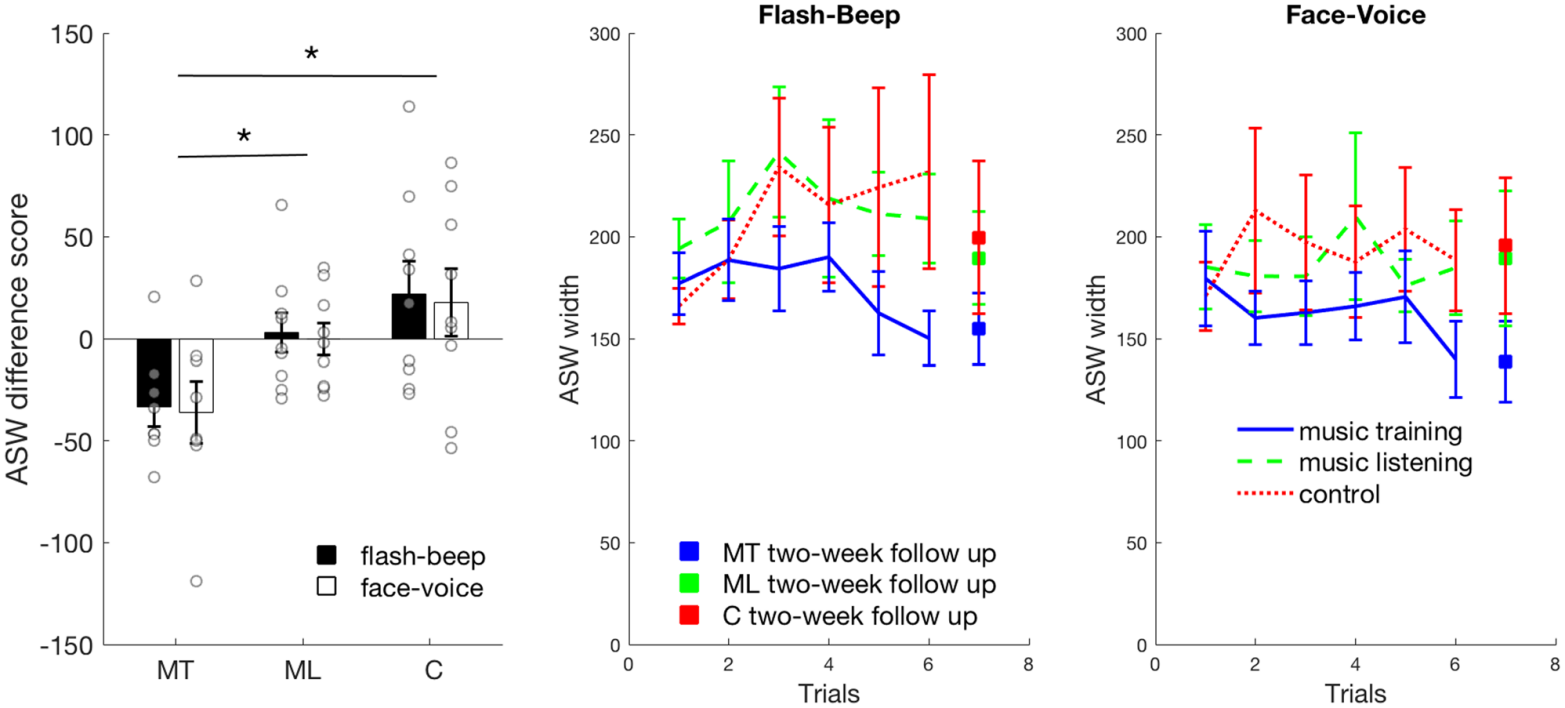
Left panel: improvement in audio-visual synchrony window (ASW difference score) was calculated as difference between the size of ASW at the last testing session and that measured at the first testing session. Negative numbers represent an improvement and a narrowing of the ASW, while positive numbers represent a worsening or enlargement of the ASW. An ASW difference score of zero indicates no difference in ASW by the end of the study. Middle and Right panel: line charts showing the group average ASW width as a function of number of trials (six bi-weekly test sessions) for flash-beep (middle panel) and face-voice (right panel) for MT (blue colour), ML (green colour), and C (red colour) group. The solid squares represent the group average ASW width at the two-week follow-up after the training ended. MT = music training group; ML = music listening group; C = control group. Error bars represent errors standard of the means and circle represent the individual data. *p < 0.05. See also Fig. S3 in the supplemental materials for participants’ individual ASW measures as a function of number of trials and for a regression analysis of the data when considering the different sessions.

To determine whether there was an effect of music training on the size of ASW in the MT group compared to the ML and C groups, we first calculated the difference in ASW score, which represents the difference between the measure of ASW at the last testing session and that obtained at the first testing session (in most cases this was calculated by ASW week11-week1). Next, we identified and removed 5 outlier values based on Boxplots (see Fig. S1 in the supplementary material). To test for group differences in ASW after the first trial (at baseline before the training started), a mixed factorial ANOVA was carried out on the ASW measures before training with group (music training, music listening and control) as the between-subjects factor and stimulus type (flash-beep and face-voice) as the within-subjects factor. This analysis was carried out for the participants entered in the main analysis (i.e., after eliminating outliers) but also returned the same results when all participants data were considered. The results revealed no significant main effect or interaction of group, *F* ≤ 0.632, *p* ≥ 0.541. Hence, all groups started from a similar measure of ASW before the starting of the training, which is also evident in Fig. 2 (middle and right panel). This also showed that participants performance in the different groups were not biased by the experimenter.

A mixed factorial ANOVA was then carried out on the ASW difference scores with group (MT, ML and C) as the between-subjects factor and stimulus type (flash-beep and face-voice) as the within-subjects factor. All the assumptions for running an ANOVA were met (see supplemental material). The results revealed a significant effect of group, *F*(2, 23) = 6.081, *p* = 0.008, *η*_*p*_^2^ = 0.346 (Fig. 2). No other factor or interaction was significant, *F* ≤ 0.195, *p* ≥ 0.663.

Post-hoc independent-samples t-tests with Bonferroni corrections were carried out to investigate the main effect of group. Results showed that by the end of the study the MT group had significantly greater reduction in ASW (more negative ASW difference scores) compared to both the ML group (*t*(15) = −3.137, *p* = 0.021, 95% CI [−60.76, −11.6], Cohen’s *d* = 1.52), and the C group (*t*(15) = −3.037, *p* = 0.024, 95% CI [−93.15, −16.32], Cohen’s *d* = 1.5). The ASW difference score for the ML group did not differ from the control group *t*(16) = −1.111, *p* = 0.849, 95% CI [−53.93, 16.83]). In line with these findings, one-sample t-tests comparing the ASW difference scores to zero (indicating no change) showed that only the MT group had a significant difference from zero, *t*(7) = −3.89, *p* = 0.006, 95% CI [−56.13, −13.68], Cohen’s d = 1.37), showing a decrease in ASW. No significant difference from zero were found for either the ML, *t*(8) = 0.172, *p* = 0.867, 95% CI [−15.78, 18.33]), nor the control group, *t*(8) = 1.325, *p* = 0.222, 95% CI [−14.68, 54.33]).

We then ran a follow-up analysis by examining whether there was an effect of testing trials for each group by running three ANOVAs with stimuli (flash-beep and face-voice) and trials (1, 2, 3, 4, 5 and 6 corresponding to week 1, 3, 5, 7, 9 and 11) as within-subjects factors. Since we had to exclude some of the individual ASW scores based on standard criteria, as explained in the Method, we carried out these ANOVAs with and without mean imputation (i.e., a common method used to fill in missing data based on the mean group performance) and they returned the same results. Therefore, we report results for the data with no mean imputation. The results revealed a significant effect of stimuli, *F*(1, 6) = 14.947, *p* = 0.008, *η*_*p*_^2^ = 0.714 and of trials, *F*(5, 30) = 4.981, *p* = 0.002, *η*_*p*_^2^ = 0.454 (Fig. 2) for the music training group, while their interaction was not significant, *F*(5, 30) = 0.339, *p* = 0.885. The significant effect of stimuli was driven by the face-voice stimulus having an overall smaller ASW (*M* = 159.64; *SD* = 52.14) than the flash-beep stimulus (*M* = 172.89; *SD* = 51.54; also see Fig. S3). Next, we ran Bonferroni corrected pairwise comparisons for the music training group and found that the effect was driven by the significant difference between the ASW scores at the end of the training (trial 6- week 11) and those at the beginning (trial 1 and 2- week 1 and 3) thus supporting the results of the first ANOVA. That is, the music training group had significant reduction in ASW by the end of the training compared to before the training started (*p* = 0.046, 95% CI [−59.36, −0.551]), and after three training sessions (*p* = 0.024, 95% CI [−64.71, −4.72]). Finally, the same ANOVAs for the music listening and control group did not return any significant results, *F* ≤ 4.483, *p* ≥ 0.079.

To further examine whether the significant reduction in ASW for the MT group persisted after the training period, we compared the ASW difference scores measured at the end of the training with those measured after a two-week period without music lessons following training (see supplemental material). Results revealed that the reduction in ASW for the MT group, seen after the training, was still maintained after the two weeks without music lessons (Fig. 2).

Next to examine whether there was a relation between AQ scores for each individual and the ASW difference scores in the MT group, we carried out a Pearson’s correlation analysis. We found a significant negative correlation for the flash-beep stimulus, *r* = −0.739, *p* = 0.036, showing that a greater reduction in ASW (or increase in audio-visual synchrony precision or acuity) was associated with greater AQ scores. However, this relation was not found for the face-voice stimulus in the music training group, *r* = 0.073, *p* = 0.864. Although this result suggests a possible relation between AQ and music training benefit on ASW, the fact that we found this relation only for the simple stimulus and these results are not corrected for multiple comparisons suggest we should consider this result with caution. No significant correlation between AQ and ASW difference scores was found for either stimuli in the music listening or control group, *r* ≤ 0.332, *p* ≥ 0.382. See Figs. S4 and S5 in the supplemental material.

### Emotion recognition

**Figure 3:**
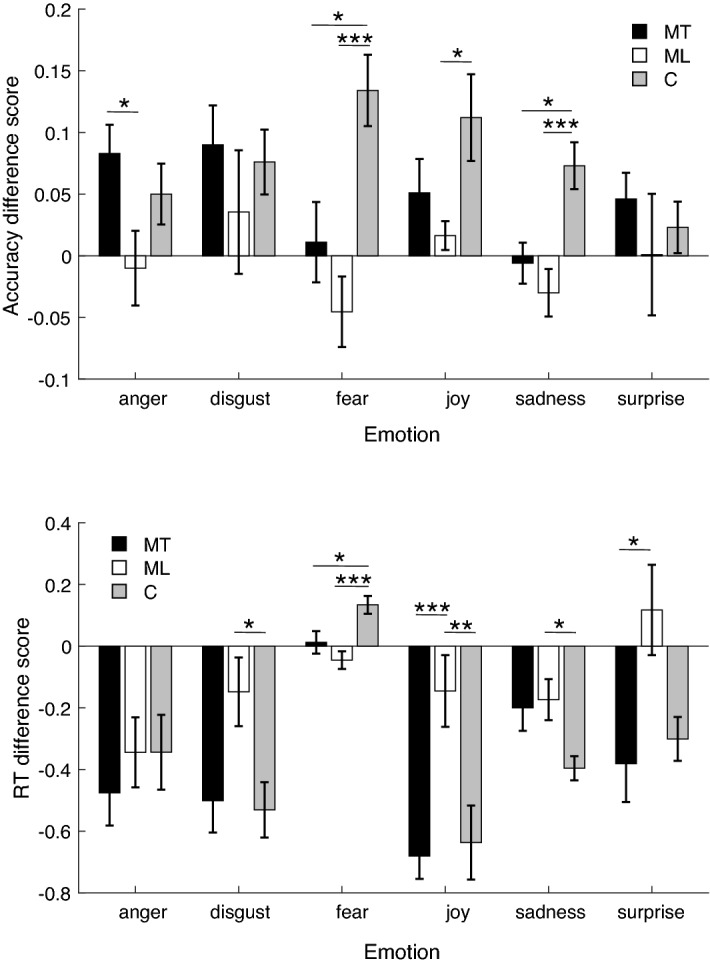
Improvement in emotion recognition accuracy (top panel) calculated as accuracy difference score (correct responses, last testing session—first testing session). Positive numbers represent an improvement in accuracy by the end of the study, while negative numbers represent a decrease in accuracy. RT difference score for correct responses (bottom panel) calculated as difference between RT at the last testing session and that measured at the first testing session. Negative numbers represent an improvement in RT by the end of the study, while positive numbers a worsening in RT. Numbers around zero indicate no difference in accuracy or RT. MT = music training group; ML = music listening group; C = control group. Error bars represent errors standard of the means. *p ≤ 0.05, **p ≤ 0.01, ***p ≤ 0.005.

To examine whether there was an effect of music training on emotion recognition accuracy and reaction time (RT) for the MT group to the ML and C groups, we first calculated the accuracy and RT difference score. This was done by calculating the difference between accuracy and RT measures at the last testing session and those at the first testing session (for a representation of mean accuracy and RT, and respective analyses, as a function of all testing sessions see Fig. S6 to Fig. S26 in the supplemental material). We then carried out a one-way ANOVA to compare the performance among the groups for the stimuli that expressed a neutral emotion (as this did not have three levels of intensity like the other emotions) and, as expected, we found no significant difference for either accuracy (proportion correct) difference scores, *F*(2, 28) = 0.976, *p* = 0.389, or RT difference scores, *F*(2, 28) = 1.354, *p* = 0.275. Then we carried out a mixed factorial ANOVA on the emotion recognition accuracy difference scores with group (MT, ML and C) as between-subjects factor and emotion category (anger, disgust, fear, joy, sadness and surprise) and emotion intensity (low, medium and high) as within-subjects factors. The analysis returned a significant effect of group, *F*(2, 28) = 5.755, *p* = 0.008, *η*_*p*_^2^ = 0.291, and a marginally significant interaction between group and emotion category, *F*(10, 140) = 1.897, *p* = 0.05, *η*_*p*_^2^ = 0.119 (Fig. 3, top panel). No other factor or interaction was significant, *F* ≤ 1.984, *p* ≥ 0.085. Pairwise comparisons, Bonferroni corrected, showed that the significant effect between groups was driven by a difference between ML and C group (*p* = 0.007, 95% CI [−0.147, −0.020]), with C group achieving a higher level of accuracy by the end of the study than the ML group. No difference was found between MT and C or MT and ML groups (*p* ≥ 0.149). In sum, although MT and C groups seem to achieve a similar level of performance at the end of the study, the ML group was consistently the one with a lower level of improvement among the three examined groups across all emotions (Fig. 3, top panel). The pairwise comparisons for group $\times$ emotion pointed to similar results and can be found in the supplemental material.

The same mixed factorial ANOVA was carried out on the calculated RT difference scores and returned similar results to the accuracy analysis. The analysis showed a significant effect of group, *F*(2, 28) = 8.268, *p* = 0.002, = 0.371, emotion category, *F*(5, 140) = 12.855, *p* < 0.001, *η*_*p*_^2^ = 0.315, and a significant interaction between group and emotion category, *F*(10, 140) = 3.073, *p* = 0.001, *η*_*p*_^2^ = 0.180 (Fig. 3, bottom panel). No other factor or interaction was significant, *F* ≤ 0.907, *p* ≥ 0.410. Bonferroni-corrected Pairwise comparisons revealed that the significant effect of group was driven by a difference between MT and ML (*p* = 0.003, 95% CI [−0.419, −0.076]), and between C and ML (*p* = 0.008, 95% CI [−0.394, −0.050]). That is, MT and C achieved faster responses by the end of the study than the ML. No difference was found between MT and C (*p* ≥ 0.999).

Similar to the results found for the accuracy data, the results for the RT data showed no clear difference between MT and C group, while the ML group was consistently the one with a lower level of improvement among the three examined groups across all emotions (Fig. 3, bottom panel). The pairwise comparisons for group $\times$ emotion pointed to similar results and can be found in the supplemental material.

Finally, to examine whether there was a relationship between the AQ score for each individual and the increase in emotion accuracy or the decrease in RT we carried out a series of Pearson’s correlation analysis. The data for accuracy and RT were collapsed across intensity as this factor had no effect, hence six correlation analyses for accuracy and six for RT were run for each group. We found only one significant positive correlation between accuracy for joy and AQ score in the MT group, *r* = 0.664, *p* = 0.036. Hence, individuals that received music training and scored higher on the AQ showed a greater improvement in accuracy when recognising joy. The same Pearson’s correlation analyses were carried out between the differences in DASS scores (DASS at the last testing session – DASS at the first testing session) and increase in emotion accuracy or decrease in RT by the end of the study, as well as between differences in PANAS scores (PANAS at the last testing session – PANAS at the first testing session calculated separately for negative and positive PANAS) and increase in emotion accuracy or decrease in RT by the end of the study. No significant correlation was found, *r* ≤ 0.602, *p* ≥ 0.066.

### DASS and PANAS

**Figure 4:**
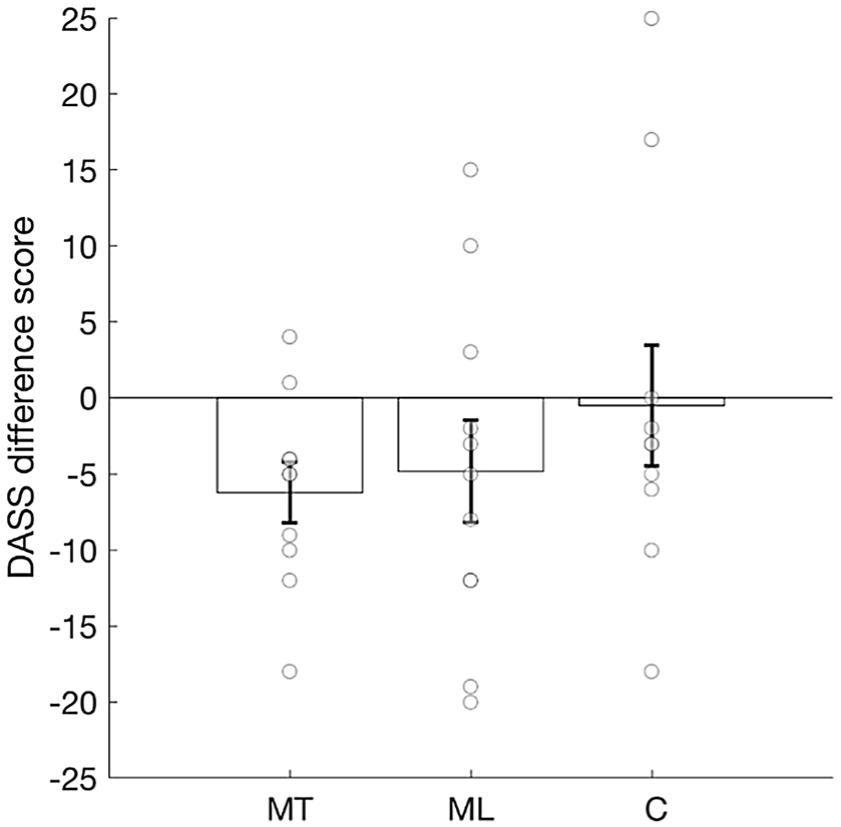
Decrease in negative emotional states of depression, anxiety and stress calculated as DASS difference score (DASS at the last testing session—DASS at the first testing session). Negative numbers represent a reduction in negative emotional states, while positive numbers represent an increase. Numbers around zero indicate no difference in negative emotional states by the end of the study. MT = music training group; ML = music listening group; C = control group. Error bars represent errors standard of the means and the circles represent the individual data.

To examine whether music training had any effect on the negative emotional states of depression, anxiety and stress, we carried out three one-sample t-tests, and found that the DASS difference score for the MT group was significantly lower than zero, (*t*(9) = −3.085, *p* = 0.013, 95% CI [−10.74, −1.65], Cohen’s *d* = 0.97; Fig. 4), indicating a significant decrease in negative emotional state for this group. No significant change in DASS was found for the ML group, *t*(10) = −1.441, *p* = 0.180, 95% CI [−12.26, 2.63], and the control group, *t*(9) = −0.126, *p* = 0.903, 95% CI [−9.48, 8.48]. A one-way ANOVA on the difference scores for the three groups revealed no significant difference among groups, *F*(2, 28) = 0.826, *p* = 0.448 (Fig. 4).

The same analysis was carried out for the PANAS positive and negative difference scores (i.e. PANAS at the first testing session – PANAS at the last testing session) (see supplemental materials Table S6, S7), but no significant change was found.

## Discussion

We carried out an RCT study to examine whether eleven weeks of piano training could improve adults’ audio-visual synchrony perception and emotion recognition abilities. We also examined whether there was a link between changes in audio-visual synchrony perception and levels of autistic traits, and in emotional recognition and levels of autistic traits as well as emotional state/mood. We randomly assigned participants to three groups: a music training group, a music listening group, or a control group with no music. We next measured their sensitivity to audio-visual synchrony (for flash and beep and face and voice clips) and their ability to recognise emotions (happiness, sadness, fear, anger, surprise, disgust, and neutrality) from facial expressions. We found that the group trained in playing the piano showed a significant improvement in sensitivity to audio-visual synchrony for both stimuli, compared to the music listening and control groups. In contrast, music training did not show any benefit beyond and above that of the control group for emotion recognition from facial expressions. Preliminary findings also suggested that participants with higher levels of autistic traits may benefit more from the training, at least for the simpler stimuli. Furthermore, only the music training group showed a significant decrease in depression, anxiety and stress scores by the end of their lessons when compared to the start of training. However, these preliminary findings should be taken with caution, given that the correlation we found between AQ scores and improvement in audio-visual synchrony perception would disappear if corrected for multiple tests and that the significant positive effect on the depression, anxiety and stress scores for the music training group was not significantly different from that of the other groups. Based on these preliminary findings, future studies should assess the possible benefit of music training for the autistic population and people with depression, anxiety or stress-related disorders, especially when presenting with alterations in processing audio-visual temporal information^42,77^.

Our findings show, for the first time, how 11 weeks of music training can significantly enhance audio-visual synchrony perception. Hence, our results support previous research showing a musician’s advantage in processing audio-visual temporal correspondence^9,15,17^. Additionally, our results show that it was the music training that delivered this advantage, rather than other factors or predispositions that cannot be excluded in cross-sectional studies. Our findings also showed that a short period of music training had a positive effect on audio-visual synchrony perception for stimuli beyond the music domain, which is consistent with findings from Lee and Noppeney^9^ and Jicol et al.^19^ cross-sectional studies showing increased ability to detect asynchrony between sinewave audio-visual speech and flash and beep stimuli in musicians compared to non-musicians. Hence, music lessons allow for near-transfer of skills in adults, pointing to a general mechanism by which music training affects audio-visual perception. The results also suggest an age unrelated educational and clinical effectiveness of music lessons, given that music lessons have been shown to have far-transfer effects in children^78,79^, but see^80,81^. Our study also showed that the improvement in audio-visual temporal processing due to music training was gradual and became evident after 11 lessons with the piano. Its unknown whether using other musical instruments may have the same benefit and require more or less time and lessons. However, current evidence suggests that training with the drums may return the same or greater benefits in enhanced abilities in audio-visual temporal processing compared to both other musicians and non-musicians^19^. Future RCT studies could compare training using musical instruments with a melodic component (e.g. the piano) to those that are purely rhythmic (e.g. the drums) to test which are more effective and faster in enhancing cognitive abilities.

In contrast, our findings showed that listening to music did not improve audio-visual synchrony perception. However, this does not mean people cannot benefit from music listening. In fact, experienced music listeners were found to perform at levels above chance performance and sometimes at the same level as musicians on tasks measuring music structure detection, musical expectancies, and music expressiveness^65,82^. However, our study did show that a few weeks of music listening did not result in a significant enhancement of multisensory abilities, in line with shorter-term training studies (two-week period) showing a significant effect on auditory and motor performances and cortical plasticity in multisensory areas for music training, but not for music listening^83–85^.

The findings for emotion recognition from facial expression did not show a facilitatory effect of music training beyond that of the control group. Although participants in the music training group became more accurate and faster at recognising the expressed emotion by the end of the study, so did participants in the control group without music, suggesting that such improvements could be merely a result of task repetition. Musicians’ emotion processing advantage has been shown primarily for music excerpts^25,26,86^ and speech prosody^10,39^, which involve the processing of sound features shared by music and speech^87,88^. Also, our results are in line with those of Correia et al.^32^ showing that musicians did not have an advantage when recognising emotions from facial expressions, and with recent evidence showing that musicians are not better than non-musicians in recognising emotions from people’s movement and gestures^11^. Taken together, the available evidence indicates that the musicians’ advantage in recognising emotion may be confined to the sound domain. This result suggests that while a short period of music lessons enhance multisensory abilities in adults, they do not enhance emotion processing of facial expressions in this population. This finding is in line with results showing a positive association between music lessons and children’s cognitive abilities, but not their social behaviour^78,89^.

Surprisingly, the music listening group showed a consistently lower level of improvement in emotion recognition than both the music training and control group. Literature has shown that listening to preferred music may be key in enhancing emotional abilities because it can activate the default mode network, which is related to core emotional memories and empathy^90^. The present study needed to match the music listening and music training group as closely as possible, such as using the same pre-selected music pieces for both groups. As our participants were non-musicians and the music training period was short, we had to select music pieces with simple melodic patterns for the training. Having to listen to simple pre-selected music repeatedly for an hour every week may have resulted in participants in the music listening group disengaging and they may not have benefitted from the music exposure. Future RCT studies could include two groups of music listening, with one listening to the pre-chosen and well-controlled music and the other to participants’ preferred music to test the effects of engagement. Since no differences in positive and negative mood were found for the three groups by the end of the study and because the music listening group showed a decrease in depression, anxiety and stress (although not significantly) by the end of the study, we believe the poor performance of the music listening group may not have been purely an effect of boredom or disengagement.

## Limitations

The current study has some limitations to be noted. The sample size we managed to recruit was not optimal due to COVID restrictions. Hence a replication of this study with a larger sample size will be needed in the future. However, the sample size in the current study was similar to, and at times, greater than previous RCT studies examining the effect of music lessons on perceptual and cognitive abilities^39,40,63^. Furthermore, despite the small sample size, the effects' size found here were generally large. Additionally, the general findings reported here using an RCT design are consistent with the findings of many cross-sectional studies^8,15^, thus determining a causal link between music training and audio-visual temporal processing.

Also, we used classical music as training materials, the same as the two RCT studies that investigated the effect of music training on emotion processing from speech prosody^39,40^. Musicians with classic music backgrounds were intensively recruited and tested in previous cross-sectional studies on the effect of music training on audio-visual^8,16^ and emotion processing^10,39^, which is why we used classical music. However, classic piano training represents only a small component of the rich and diverse musical experience. There is evidence to show that classical and jazz pianists have genre-specific neurobiological differences^91^, and training with different instruments could results in varied structural and functional brain plasticities^92,93^. That is, instrument types and music genres may affect how people’s audio-visual and emotion processing abilities could benefit from music training. Hence, these factors should be further investigated in future research by comparing the effect of music training with different music instruments on multisensory and emotion processing.

As the current study is the first to use an RCT design to investigate the effect of music training on audio-visual speech processing, we aimed to match the length of the face-voice and flash-beep stimuli to increase the comparability of music training’s effects on both stimulus types. This was done to avoid the inclusion of confounding factors linked to differences in complexity and dynamics among the two stimuli (e.g., a face pronouncing a word is not just different from a flash and beep sound because of the speech characterisation but also is different in duration and overall stimulus complexity making the synchrony judgement task more difficult)^68,69^. However, for the above reasons, the face-voice stimuli in this study were restricted to the vowel ‘o’ and had minimum lip movements, unlike other previous studies^9,16^. Hence, the current speech stimuli could only reflect the natural speech to a limited degree. Future studies are needed to examine whether music training can also benefit audio-visual temporal processing for more complex speech such as words and sentences.

We did not include measures to document participants’ musical achievement at the end of the study, similarly to previous RCT studies that have involved musical training^39,40^. However, a more recent study investigating the cognitive benefits of music training has included evaluations of musical abilities and learning outcomes at multiple time points^94^, which offers an additional check for the design effectiveness. Future studies should take this into consideration and include music achievement measures in the study design.

We measured participants’ level of autistic traits using the self-report AQ^46^, but we did not include individuals diagnosed with ASD. Thus, the results from the present study do not offer insight into the effect of music training on the autistic population. Although we found suggestive evidence that people with higher levels of autistic traits could benefit more from music training in audio-visual temporal processing, future lines of research that apply a similar RCT design involving samples of individuals with a diagnosis of ASD are necessary to determine the impact of music training on the autistic population.

Finally, we could not further investigate the underlying mechanisms of how music training could enhance audio-visual temporal processing with the current design and methods. We did not include any neuroimaging or neurophysiological measures and thus could not directly compare our findings with Lee and Noppeney^9,16^ and Petrini et al.^8,17^ to consider whether differences in brain activity and connectivity shown by these studies can be detected after a few music lessons. Since all previous studies adopted a cross-sectional design, the current study is the first to establish a causal relationship between music training and enhanced audio-visual temporal processing ability. Therefore, based on our findings, the reasonable next step is to explore the underlying mechanism for this causal relationship.

## Conclusion

In conclusion, the current study presents important evidence on the causal link between music training and audio-visual temporal processing. Eleven weeks of piano training can improve people’s audio-visual asynchrony detection and possibly their level of depression, anxiety and stress, but not their ability to recognise facial expressions. We show that people with higher autistic traits may benefit from a short period of music training when processing audio-visual temporal information and recognising some emotions like joy. However, more research is needed to achieve clearer conclusions about these preliminary results.

## Supplementary Information

Supplementary Information 1.

Supplementary Video 1.

Supplementary Video 2.

Supplementary Video 3.

Supplementary Video 4.

Supplementary Video 5.

Supplementary Video 6.

Supplementary Video 7.

Supplementary Video 8.

Supplementary Video 9.
