# Supplementary material for: An RCT study showing few weeks of music lessons enhance audio-visual temporal processing
Source: Sci Rep. 2022 Nov 22;12:20087. doi: 10.1038/s41598-022-23340-4 (PMC9684138; doi:10.1038/s41598-022-23340-4)
Supplement: Supplementary file 1 — Supplementary Information 1. [file 41598_2022_23340_MOESM1_ESM.docx]

An RCT study showing few weeks of music lessons enhance audio-visual temporal processing

Yuqing Che^1^, Crescent Jicol^2^, Chris Ashwin^1^, Karin Petrini^1^

^1^Department of Psychology, University of Bath, Claverton Down, Bath, BA2 7AY, UK

^2^Department of Computer Science, University of Bath, Claverton Down, Bath, BA2 7AY, UK

**Corresponding Author:**

Yuqing Che

PhD candidate

Department of Psychology

University of Bath

Claverton Down

Bath

BA2 7AY

United Kingdom

Email: [s_r2@hotmail.com](mailto:s_r2@hotmail.com);

yc2210@bath.ac.uk


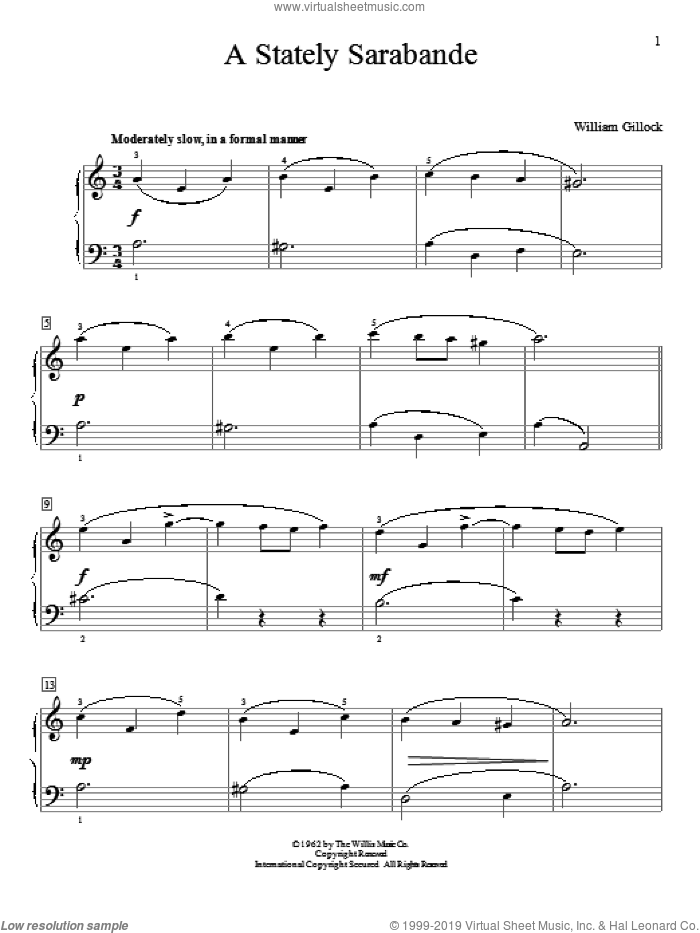


Figure S1. Example of music sheet ‘A stately Sarabande’ used for the music lessons.

**Teaching curriculum for music training lessons**

Each music training session included two segments. The first 20-minute segment was dedicated to finger exercises using the Hanon: the Virtuoso Pianist, Book 1: in Sixty Exercises for the Piano. This version of Hanon consists of 20 finger exercise pieces with progressive complexity. Each participant in the music training group started with the Hanon exercise No.1 and proceeded in sequential order. They were guided to proceed to the next exercise once they could play the former one fluently. The second segment consisted of learning songs from the ABRSM 2017-2018 piano grade one exam list for 40 minutes. We chose five piano pieces from the exam list for their balanced length and complexity, making them suitable for beginners. Participants learned these pieces in the order presented below. They proceeded to the next song once they could play the former one correctly and fluently.

1. William Gillock A Stately Sarabande. Classic Piano Repertoire (Elementary)
2. Johann Christian Bach Aria in F, BWV Anh. II 131
3. Giuseppe Verdi La donna è mobile (from Rigoletto)
4. Bryan Kelly Gypsy Song: No. 6 from A Baker’s Dozen
5. Traditional American Folk Song: When the saints go marching in

All training sessions were carried out on a one-to-one basis. During the 20-minute segment, the teacher first introduced the key technique and focus of the chosen finger exercise. For example, the Hanon exercise No.1 is designed to help stretch between the fourth and fifth fingers in both hands to build independence. It is critical to lift each finger high and with precision during the practice so that each note can be heard distinctly. The same finger movement pattern is repeated throughout each Hanon finger exercise (with one note higher or lower each time). Therefore, the teacher illustrated the pattern with the first music measure (bar) then guided the participant to practice the pattern one hand at a time. Once they were familiarised with the pattern and could play one music measure accurately, participants were then encouraged to play the entire finger exercise with both hands at an even, consistent speed. During the second segment, the teacher first played the chosen piece once and introduced the musical genre and character. For example, the Aria in F by Bach is a baroque era classical music that has fewer requirements for performance details. Participants were then encouraged to use a few words to describe their impression of the piece, e.g., what did this music remind them of, and what kind of emotion did the music convey? After that, the teacher divided the music score into smaller sections by music sentence (one music sentence usually consists of six to eight measures) and taught the participants to play each sentence one hand at a time. This teaching approach is often referred to as ‘hands separate practice’, which is commonly used to teach beginners as an acquiring technique. Once participants could play each hand’s part fluently, they were then encouraged to try to combine the two hands together. On the first attempt, all participants found the cooperation of two hands challenging. Therefore, the teacher trained them to coordinate one hand at a time with the teacher playing either the left or right hand’s notes while the student played the other hand’s matching notes. During this process, participants were instructed to match the teacher's speed and pay extra attention to the music flow on both hands to form a unified representation of the song. The teacher individualised the length of the matching-play-with-teacher exercise based on each participant's speed of progression. Participants required around five to ten repetitions of the matching exercise for each hand for each music sentence. Then, the teacher would ask the participant to coordinate the two hands again. Usually, at this stage, participants could match the notes of both hands at a slow speed. They were encouraged to practice one music sentence until a satisfactory level was achieved . This process was repeated until the participant could play the piece successfully with both hands. They were then given more time to practice independently during the music lesson while the teacher sat next to them and provided guidance when necessary. By the end of study, participants in the music training group learned to play at least ten Hanon finger practice sequences (range 10-15) and three songs (range 3-5) from the 2017-2018 Grade 1 ABRSM piano exam list fluently.

**List of songs for music listening sessions**

We made a one-hour loop playlist to match the music training group's learning content for the music listening sessions. This playlist consisted of all 18 piano songs selected as 2017-2018 ABRSW grade 1 piano exam pieces, including the five songs chosen for the music training session in this study. The entire playlist was around 15 minutes long and looped four times during each listening session. It can be viewed and downloaded from the ABRSW website (https://www.abrsmdownloads.org/Piano_2017_&_2018).

Table S1. Testing and training timetable for the music training group.

|  | **Activities** |
| --- | --- |
| **Week 1**  (2 hrs) | AQ, PANAS, DASS |
|  | SJ, ER |
|  | Piano training for 1 hour |
| **Week 2** | Piano training for 1 hour |
| **Week 3**  (2 hrs) | Piano training for 1 hour |
|  | SJ, ER |
|  | PANAS, DASS |
| **Week 4** | Piano training for 1 hour |
| **Week 5**  (2 hrs) | Piano training for 1 hour |
|  | SJ, ER |
|  | PANAS, DASS |
| **Week 6** | Piano training for 1 hour |
| **Week 7**  (2 hrs) | Piano training for 1 hour |
|  | SJ, ER |
|  | PANAS, DASS |
| **Week 8** | Piano training for 1 hour |
| **Week 9**  (2 hrs) | Piano training for 1 hour |
|  | SJ, ER |
|  | PANAS, DASS |
| **Week 10** | Piano training for 1 hour |
| **Week 11**  (2 hrs) | Piano training for 1 hour |
|  | SJ, ER |
|  | PANAS, DASS |
| **Week 12** | No need to come |
| **Week 13**  (<1 hr) | AQ, PANAS, DASS |
|  | SJ, ER |

**AQ:** Adult Autism Spectrum Quotient

**PANAS:** Positive and Negative Affect Schedule

**DASS:** Depression Anxiety Stress Scale

**SJ:** Simultaneity Judgement task on computer

**ER:** Emotion Recognition task on computer

Table S2. Testing and training timetable for the music listening group.

|  | **Activities** |
| --- | --- |
| **Week 1**  (2 hrs) | AQ, PANAS, DASS |
|  | SJ, ER |
|  | Piano listening for 1 hour |
| **Week 2** | Piano listening for 1 hour |
| **Week 3**  (2 hrs) | Piano listening for 1 hour |
|  | SJ, ER |
|  | PANAS, DASS |
| **Week 4** | Piano listening for 1 hour |
| **Week 5**  (2 hrs) | Piano listening for 1 hour |
|  | SJ, ER |
|  | PANAS, DASS |
| **Week 6** | Piano listening for 1 hour |
| **Week 7**  (2 hrs) | Piano listening for 1 hour |
|  | SJ, ER |
|  | PANAS, DASS |
| **Week 8** | Piano listening for 1 hour |
| **Week 9**  (2 hrs) | Piano listening for 1 hour |
|  | SJ, ER |
|  | PANAS, DASS |
| **Week 10** | Piano listening for 1 hour |
| **Week 11**  (2 hrs) | Piano listening for 1 hour |
|  | SJ, ER |
|  | PANAS, DASS |
| **Week 12** | No need to come |
| **Week 13**  (<1 hr) | AQ, PANAS, DASS |
|  | SJ, ER |

**AQ:** Adult Autism Spectrum Quotient

**PANAS:** Positive and Negative Affect Schedule

**DASS:** Depression Anxiety Stress Scale

**SJ:** Simultaneity Judgement task on computer

**ER:** Emotion Recognition task on computer

Table S3. Testing and training timetables for the control group.

|  | **Activities** |
| --- | --- |
| **Week 1**  (2 hrs) | AQ, PANAS, DASS |
|  | Study for 1 hour |
|  | SJ, ER |
| **Week 2** | No need to come |
| **Week 3**  (2 hrs) | Study for 1 hour |
|  | SJ, ER |
|  | PANAS, DASS |
| **Week 4** | No need to come |
| **Week 5**  (2 hrs) | Study for 1 hour |
|  | SJ, ER |
|  | PANAS, DASS |
| **Week 6** | No need to come |
| **Week 7**  (2 hrs) | Study for 1 hour |
|  | SJ, ER |
|  | PANAS, DASS |
| **Week 8** | No need to come |
| **Week 9**  (2 hrs) | Study for 1 hour |
|  | SJ, ER |
|  | PANAS, DASS |
| **Week 10** | No need to come |
| **Week 11**  (2 hrs) | Study for 1 hour |
|  | SJ, ER |
|  | PANAS, DASS |
| **Week 12** | No need to come |
| **Week 13**  (<1 hr) | AQ, PANAS, DASS |
|  | SJ, ER |

**AQ:** Adult Autism Spectrum Quotient

**PANAS:** Positive and Negative Affect Schedule

**DASS:** Depression Anxiety Stress Scale

**SJ:** Simultaneity Judgement task on computer

**ER:** Emotion Recognition task on computer

**Basic Information Questionnaire**

**Thank you for your participation in this research project, your contribution has been very important to the project. Please answer the following questions according to your real situation.**

**Your full name:**

**Gender:** □Male □Female □Other

**Date of Birth:**

**Age:**

**Nationality:**

**I am:** □Right-handed □Left-handed

**How often do you listen to music every week? __________**

1. Less than 1 hr. 2) 1-3 hr. 3) 3-5 hr. 4) 5-7 hr.

5) 7-9 hr. 6) 9-11 hr. 7) 11-13 hr. 8) More than 14hr.

**Have you received any kind of music training before**: □Yes □No

If choose Yes, please specify:

**Your email address:**

**Your contact number:**

**Please tick the box if you do not want to be contacted by:**

□Message □Phone-call

Your data will be used for academic purposes only, will be kept confidential, will be completely anonymous (it will not be possible to link any set of data with any particular individual), and securely stored. If you are unhappy in any way about the use of your data you maintain the right to withdraw the responses you have provided in this study. Please feel free to ask the experimenter any additional questions you may have regarding this study, and to contact YC via email.


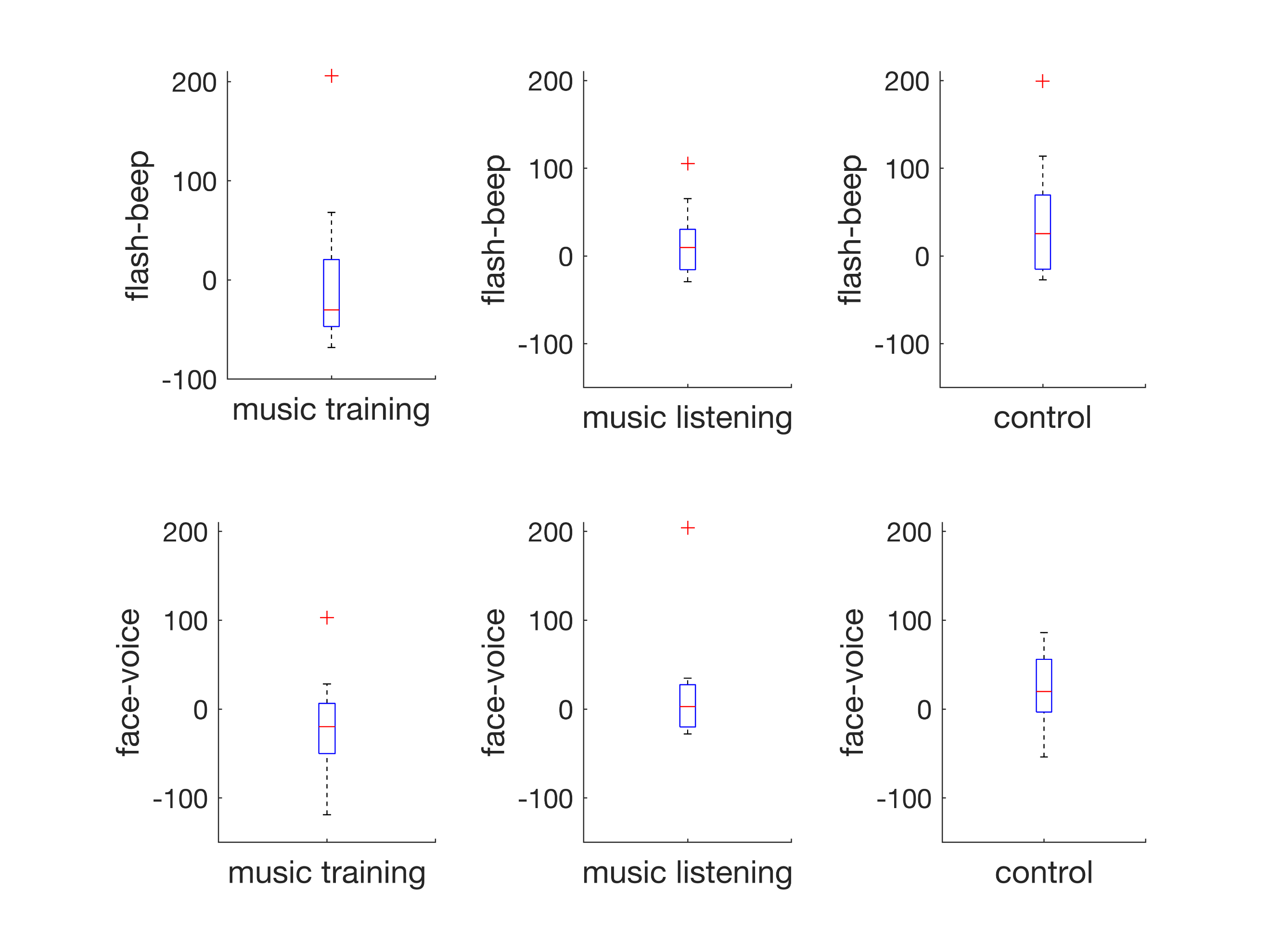


Figure S2. The boxplots show the median, interquartile range and the outliers for the ASW difference scores, which is the difference between the ASW at the last testing session and the ASW at the first testing session. Left panels: the music training group ASW difference scores for the flash-beep stimuli on the top row and for the face-voice stimuli on the bottom row. Central panels: the music listening group ASW difference scores for the flash-beep stimuli on the top row and for the face-voice stimuli on the bottom row. Right panels: the control group ASW difference scores for the flash-beep stimuli on the top row and for the face-voice stimuli on the bottom row. The cross represents the outliers. A total of 5 outliers (one in each group for the flash-beep stimuli, one in the music training group and one in the music listening group for the face-voice stimuli) were identified and removed.

Table S4. Autism Quotient (AQ) results (MT = music training; ML = music listening; C = control)

| **Participant Number** | **Group** | **AQ Week 1** | **AQ Week 13** |
| --- | --- | --- | --- |
| 1 | MT | 29 | 29 |
| 6 | MT | 14 | 13 |
| 10 | MT | 23 | 21 |
| 11 | MT | 24 | 28 |
| 14 | MT | 11 | 11 |
| 19 | MT | 24 | 28 |
| 18 | MT | 24 | 24 |
| 22 | MT | 6 | 12 |
| 26 | MT | 20 | 23 |
| 35 | MT | 7 | 7 |
| 2 | ML | 16 | 16 |
| 4 | ML | 16 | 18 |
| 9 | ML | 18 | 23 |
| 12 | ML | 9 | 6 |
| 20 | ML | 32 | 33 |
| 24 | ML | 12 | 17 |
| 21 | ML | 26 | 21 |
| 25 | ML | 20 | 20 |
| 32 | ML | 18 | 17 |
| 34 | ML | 23 | 24 |
| 36 | ML | 15 | 21 |
| 5 | C | 21 | 17 |
| 8 | C | 9 | 11 |
| 13 | C | 21 | 26 |
| 15 | C | 23 | 22 |
| 16 | C | 12 | 19 |
| 17 | C | 19 | 19 |
| 23 | C | 9 | 7 |
| 28 | C | 23 | 22 |
| 31 | C | 23 | 23 |
| 33 | C | 23 | 27 |

**Analysis of the AQ results**

The AQ was measured twice, once at the beginning of the study (AQ1) and then again at the end of the study (AQ2). To determine the level of consistency between the two AQ measures, we carried out a Pearson’s correlation analysis. We found a significant positive correlation between AQ1 and AQ2, r = .895, *p* < .001, which showed a high test-retest reliablity. Therefore, the mean score of the two AQ measures was used as the final AQ score for the analyses. A one-way ANOVA was carried out to compare the AQ scores between three groups (MT, ML, and C) and showed no significant difference F(2, 28) = 0.008, *p* = .992.

Table S5. Depression Anxiety Stress Scales (DASS) results (MT = music training; ML = music listening; C = control)

| **Participant Number** | **Group** | **Week1** | **Week3** | **Week5** | **Week7** | **Week9** | **Week11** | **Week13** |
| --- | --- | --- | --- | --- | --- | --- | --- | --- |
| 1 | MT | 13 | 12 | 21 | 9 | 12 | 9 | 11 |
| 6 | MT | 23 | 7 | 9 | 16 | 25 | 5 | 9 |
| 10 | MT | 5 | 2 | 1 | 35 | 4 | 9 | 29 |
| 11 | MT | 17 | 9 | 8 | 10 | 10 | 8 | 17 |
| 14 | MT | 4 | 12 | 1 | 0 | 0 | 0 | 0 |
| 19 | MT | 8 | 5 | 4 | 8 | 6 | 3 | 0 |
| 18 | MT | 12 | 20 | 14 | 7 | 13 | 13 | 6 |
| 22 | MT | 10 | 6 | 4 | 3 | 4 | 5 | 7 |
| 26 | MT | 17 | 13 | 18 | 16 | 15 | 5 | 21 |
| 35 | MT | 20 | 21 | 35 | 30 | 19 | no data | no data |
| 2 | ML | 7 | 8 | 5 | 1 | 1 | 4 | 9 |
| 4 | ML | 25 | 22 | 30 | 18 | 23 | 13 | 17 |
| 9 | ML | 22 | 25 | 23 | 15 | 22 | 32 | 14 |
| 12 | ML | 2 | 1 | 0 | 0 | 0 | 0 | 0 |
| 20 | ML | 22 | 18 | 25 | 30 | 48 | 25 | 36 |
| 24 | ML | 22 | 16 | 23 | 8 | 4 | 10 | 4 |
| 21 | ML | 5 | 2 | 0 | 2 | 0 | 0 | 8 |
| 25 | ML | 10 | 18 | 7 | 10 | 3 | 25 | 11 |
| 32 | ML | 8 | 0 | 0 | 0 | 0 | 0 | 0 |
| 34 | ML | 31 | 11 | 14 | 12 | 10 | 11 | 11 |
| 36 | ML | 38 | 32 | 21 | 9 | 22 | 19 | no data |
| 5 | C | 24 | 13 | 4 | 5 | 6 | 6 | 5 |
| 8 | C | 4 | 0 | 2 | 3 | 4 | 4 | 4 |
| 13 | C | 7 | 9 | 4 | 3 | 1 | 5 | 4 |
| 15 | C | 15 | 16 | 11 | 10 | 3 | 5 | 7 |
| 16 | C | 5 | 13 | 28 | 16 | 23 | 22 | 25 |
| 17 | C | 21 | 46 | 23 | 21 | 26 | 18 | 1 |
| 23 | C | 3 | 2 | 4 | 13 | 3 | 0 | 7 |
| 28 | C | 10 | 4 | 0 | 0 | 2 | 4 | 2 |
| 31 | C | 12 | 8 | 20 | 29 | 23 | 37 | 21 |
| 33 | C | 17 | 7 | 8 | 10 | 10 | 12 | 9 |

Table S6. Positive and Negative Affect Schedule (PANAS) results for positive emotions

| **Participant Number** | **Group** | **Week1** | **Week3** | **Week5** | **Week7** | **Week9** | **Week11** | **Week13** |
| --- | --- | --- | --- | --- | --- | --- | --- | --- |
| 1 | MT | 28 | 17 | 28 | 28 | 33 | 30 | 22 |
| 6 | MT | 36 | 40 | 39 | 40 | 39 | 41 | 24 |
| 10 | MT | 21 | 16 | 13 | 13 | 10 | 14 | 12 |
| 11 | MT | 18 | 28 | 28 | 24 | 45 | 42 | 34 |
| 14 | MT | 30 | 40 | 35 | 35 | 38 | 35 | 21 |
| 19 | MT | 24 | 13 | 20 | 20 | 22 | 26 | 22 |
| 18 | MT | 32 | 27 | 21 | 26 | 24 | 12 | 24 |
| 22 | MT | 27 | 31 | 23 | 22 | 21 | 23 | 17 |
| 26 | MT | 29 | 29 | 24 | 37 | 14 | 16 | 15 |
| 35 | MT | 38 | 40 | 45 | 43 | 31 | No data | No data |
| 2 | ML | 36 | 23 | 19 | 28 | 22 | 21 | 30 |
| 4 | ML | 29 | 40 | 26 | 27 | 29 | 21 | 32 |
| 9 | ML | 28 | 30 | 24 | 28 | 22 | 21 | 38 |
| 12 | ML | 34 | 30 | 29 | 30 | 33 | 35 | 30 |
| 20 | ML | 28 | 24 | 21 | 17 | 15 | 23 | 20 |
| 24 | ML | 38 | 26 | 25 | 28 | 32 | 30 | 28 |
| 21 | ML | 33 | 33 | 36 | 34 | 32 | 41 | 29 |
| 25 | ML | 32 | 36 | 34 | 27 | 32 | 24 | 33 |
| 32 | ML | 49 | 50 | 45 | 45 | 44 | 46 | 47 |
| 34 | ML | 22 | 31 | 28 | 26 | 36 | 23 | 31 |
| 36 | ML | 21 | 17 | 27 | 25 | 27 | 30 | No data |
| 5 | C | 13 | 14 | 19 | 19 | 20 | 20 | 16 |
| 8 | C | 27 | 26 | 13 | 16 | 15 | 17 | 15 |
| 13 | C | 27 | 35 | 27 | 35 | 30 | 30 | 34 |
| 15 | C | 40 | 28 | 21 | 19 | 25 | 11 | 26 |
| 16 | C | 38 | 32 | 29 | 32 | 27 | 28 | 22 |
| 17 | C | 34 | 19 | 34 | 35 | 40 | 37 | 37 |
| 23 | C | 36 | 42 | 33 | 28 | 33 | 40 | 41 |
| 28 | C | 40 | 42 | 40 | 37 | 44 | 44 | 44 |
| 31 | C | 28 | 31 | 22 | 24 | 27 | 25 | 26 |
| 33 | C | 19 | 16 | 19 | 20 | 17 | 14 | 17 |

Table S7. Positive and Negative Affect Schedule (PANAS) results for negative emotions (MT = music training; ML = music listening; C = control)

| **Participant Number** | **Group** | **Week1** | **Week3** | **Week5** | **Week7** | **Week9** | **Week11** | **Week13** |
| --- | --- | --- | --- | --- | --- | --- | --- | --- |
| 1 | MT | 21 | 13 | 16 | 13 | 12 | 13 | 20 |
| 6 | MT | 11 | 19 | 14 | 21 | 31 | 17 | 10 |
| 10 | MT | 11 | 13 | 12 | 24 | 12 | 10 | 22 |
| 11 | MT | 10 | 10 | 10 | 10 | 10 | 10 | 16 |
| 14 | MT | 11 | 12 | 13 | 13 | 12 | 10 | 10 |
| 19 | MT | 10 | 10 | 10 | 10 | 10 | 10 | 10 |
| 18 | MT | 12 | 10 | 11 | 11 | 11 | 10 | 11 |
| 22 | MT | 12 | 10 | 11 | 10 | 13 | 13 | 15 |
| 26 | MT | 12 | 14 | 10 | 10 | 10 | 10 | 10 |
| 35 | MT | 13 | 12 | 12 | 11 | 11 | No data | No data |
| 2 | ML | 19 | 14 | 18 | 10 | 10 | 15 | 12 |
| 4 | ML | 19 | 39 | 25 | 14 | 22 | 11 | 12 |
| 9 | ML | 22 | 30 | 21 | 27 | 31 | 26 | 15 |
| 12 | ML | 11 | 10 | 10 | 10 | 10 | 10 | 10 |
| 20 | ML | 20 | 20 | 26 | 33 | 36 | 25 | 36 |
| 24 | ML | 23 | 29 | 34 | 10 | 12 | 21 | 13 |
| 21 | ML | 10 | 10 | 10 | 10 | 10 | 10 | 22 |
| 25 | ML | 10 | 24 | 21 | 11 | 11 | 21 | 20 |
| 32 | ML | 14 | 19 | 16 | 14 | 18 | 10 | 15 |
| 34 | ML | 10 | 10 | 10 | 14 | 10 | 16 | 11 |
| 36 | ML | 13 | 37 | 18 | 13 | 19 | 14 | No data |
| 5 | C | 13 | 12 | 10 | 10 | 10 | 12 | 9 |
| 8 | C | 13 | 10 | 11 | 10 | 10 | 10 | 10 |
| 13 | C | 12 | 15 | 19 | 13 | 11 | 12 | 12 |
| 15 | C | 12 | 12 | 11 | 12 | 23 | 20 | 10 |
| 16 | C | 10 | 15 | 37 | 23 | 22 | 35 | 22 |
| 17 | C | 14 | 37 | 13 | 23 | 12 | 13 | 12 |
| 23 | C | 10 | 10 | 13 | 15 | 11 | 10 | 10 |
| 28 | C | 10 | 13 | 11 | 11 | 10 | 13 | 13 |
| 31 | C | 13 | 11 | 22 | 29 | 26 | 31 | 26 |
| 33 | C | 10 | 12 | 11 | 15 | 12 | 14 | 10 |

**Additional analyses, figures and check of assumptions**

*ASW*

A mixed factorial ANOVA was carried out on the ASW difference scores (difference in ASW after the study) with group (music training, music listening and control) as the between-subjects factor and stimulus type (flash-beep and face-voice) as the within-subjects factor. A Shapiro-Wilk test of normality showed no significant deviation from normality in the data for all groups and stimuli condition (*p* ≥ .308). Furthermore, Levene’s tests showed equality of variance across groups (*p* ≥ .160), showing that all key assumptions for the test were met.

Plots representing the individual data for each participant in each group and for each stimulus separately can be seen in Figure S3. Here we report a different way of analysing the data and examining the effect of groups and trials while dealing with missing data. We fitted each individual performance (ASW) with a linear regression and identified the participants in each group who improved (showed reduction in ASW) and those that did not by using the slope of the regression analysis (e.g., those who showed reduction in ASW = negative slope). This allowed us to get this information for all the participants even when they had some missing ASW because of exclusions criteria. We thus first obtained the percentage of participants that showed improvement in each group and stimulus type when considering all trials, and secondly we obtained the average ASW at each trial across all the participants that did show an improvement in each group and each stimulus type. We then run a multiple regression analysis on the data that did have a data point for each trial. The multiple regression analysis with trials and stimuli as predictors and ASW as outcome showed that trial was a significant predictor of mean ASW (*p* = .01) only for the music training group (Figure S3 top right panel), with a significant regression equation, *F*(2, 9) = 12.220, *p* < .003, $R^{2}$= .731. Participants’ ASW decreased by 6.82 ms at each trial in this group. Stimulus type was also a significant predictor of ASW (*p* = .005) for the music training group with the flash-beep stimuli having overall a larger ASW than face-voice stimuli of 26.53 ms. No significant regression equation was found for the control group, *F*(2, 9) = 3.371, *p* = .081, $R^{2}$= .428, while for the ML group a significant regression equation was found, *F*(2, 9) = 9.054, *p* = .007, $R^{2}$= .668. However, only the predictor stimulus type was significant for this group (*p* = .002), indicating that flash-beep stimuli had a larger ASW than face-voice stimuli. These findings replicated those reported in the main manuscript using a different exclusion criteria and different way of dealing with missing data, thus increasing the confidence in our findings despite the small sample size.


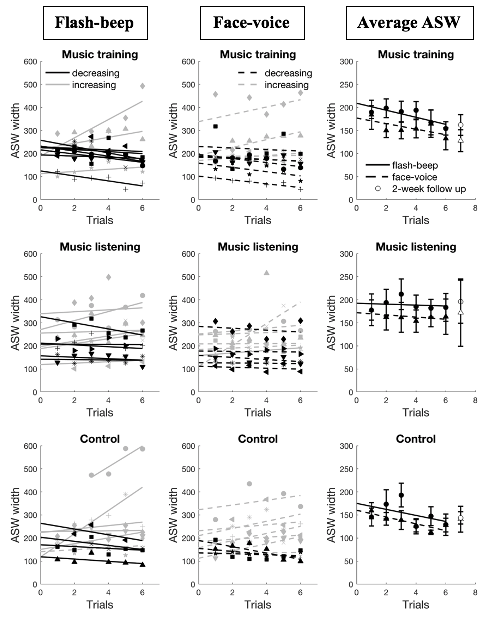


Figure S3. Left panels: Individual ASW measures as a function of number of trials (6 bi-weekly testing sessions) for the flash-beep stimulus for the music training group on top, the music listening group in the middle and the control group at the bottom. Central panels: Individual ASW measures as a function of number of trials for the face-voice stimulus for the music training group on top, the music listening group in the middle and the control group at the bottom. The black lines represent the individuals in each group that decreased their ASW with trials and the grey lines the individuals in each group that increased their ASW with trials. Right panels: Average ASW for the individuals that showed a decrease in ASW width (represented by the black lines in the left and central panels). The average ASW is shown as a function of number of trials for flash-beep (circles) and face-voice (triangles) for the music training group on top, the music listening group in the middle and the control group at the bottom. The error bars represent the standard errors of the means, and the white circle the follow-up measurement of ASW. Three multiple regression analyses were run for the average ASW data shown in the right panels for music training, music listening and control group. Trials or testing sessions (from first to last, range 1 to 6) and stimulus type (flash-beep and face-voice) were used as predictors and mean ASW as outcome.

A mixed factorial ANOVA was also carried out to analyse whether the ASW difference scores changed after a two-week follow-up period, with group (music training, music listening and control) as the between-subjects factor, stimulus type (flash-beep and face-voice) and testing session (in most cases this reflected a comparison between ASW in week 11 and in week 13) as the within-subjects factor. This analysis was carried out for the participants entered in the main analysis (i.e., after eliminating outliers), but it also produced the same results when all participants data were considered. The results revealed no significant main or interaction effect for trial/testing session, *F* ≤ 1.023, *p* ≥ .378. Hence, no significant change in ASW was found for any group after the two-week follow-up period.

*Autism Spectrum Quotients (AQ)*

The figure below refers to the correlation analyses between AQ and ASW reduction presented in Results section of the main manuscript.


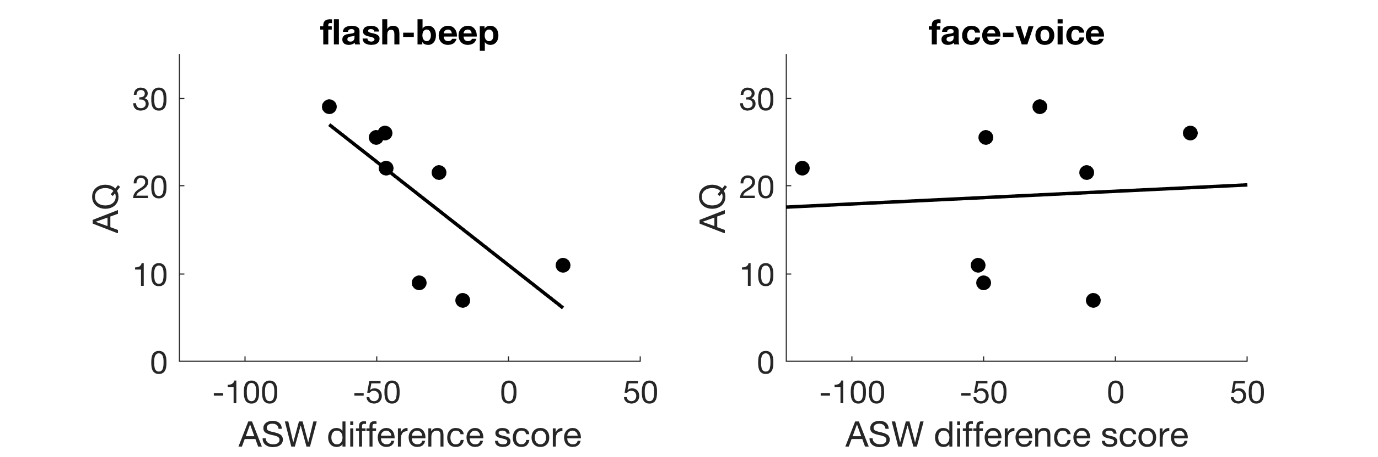


Figure S4. Relation between individuals’ ASW difference score (last testing session – first testing session) and AQ scores within the MT group. The left panel shows the relation for the flash-beep stimulus while the right panel for the face-voice stimulus.

We carried out a second Pearson’s analysis between AQ scores and individuals’ ASW difference score after considering the changes in ASW as a function of all six measurements of ASW, i.e., for the participants that showed a gradual improvement in ASW in the music training group in Figure S3. We achieved similar results (Figure S5) in that we found a significant negative correlation for the flash-beep stimulus, *r* (7)= -.810, *p* = .027 (Figure S5, left panel), but not for the face-voice stimulus.


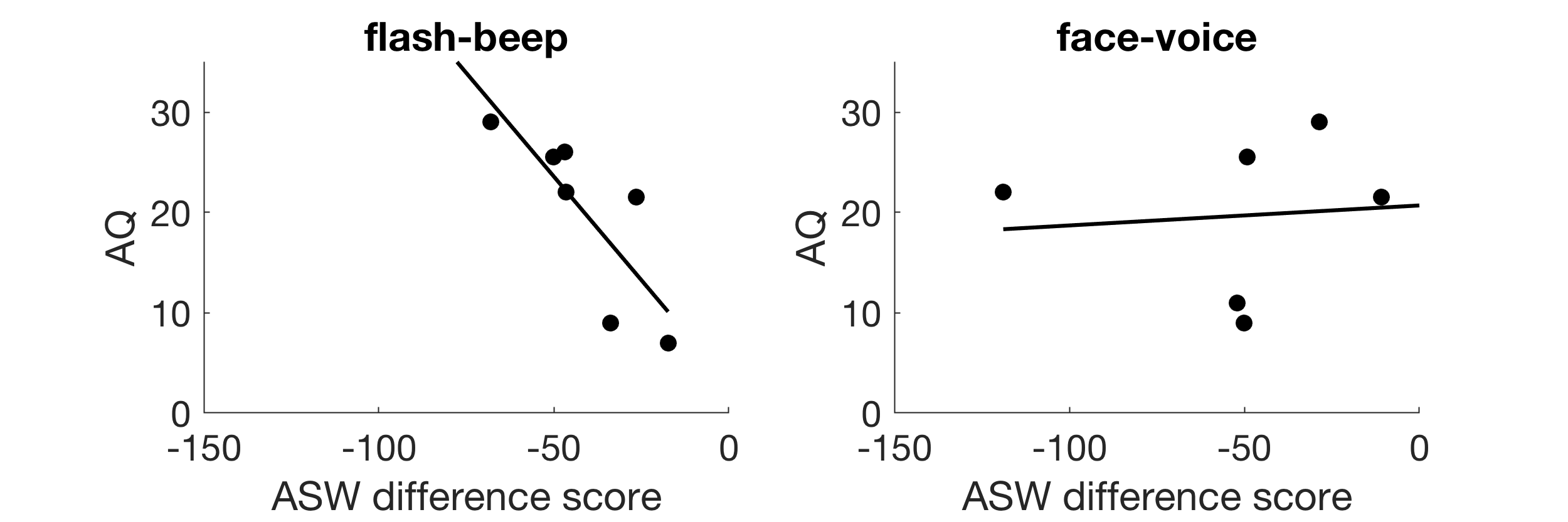


Figure S5. Relation between individuals’ ASW difference score (ASW from last testing session – ASW from first testing session) and AQ scores for the participants that showed a gradual improvement in ASW in the music training group (refer to the bold lines in Figure S3 top left and middle panel). The left panel shows the relation for the flash-beep stimulus while the right panel for the face-voice stimulus.

Such findings suggested that a greater reduction in ASW (or increase in audio-visual synchrony precision or acuity) was associated with greater AQ scores. Hence, individuals that scored higher on the AQ improved their ASW more with music lessons than individuals that scored lower on the AQ for the simpler stimulus type.

**Additional figures and analyses of accuracy and reaction time for the emotion recognition task**

**Post hoc for group x emotion**

Group differences in the individual emotion categories were tested with Bonferroni corrected pairwise comparisons. Results revealed no significant differences between the groups for disgust and surprise (*p* ≥ .962). For anger, the MT group was marginally more accurate than the ML group (*p* = .058, 95% CI [-.002, .188]) by the end of the study, but not more accurate than the control group (*p > .*999). For fear, the C group was more accurate than both the ML (*p* = .001, 95% CI [.072, .287]) and the MT group (*p = .*026, 95% CI [.012, .233]). For joy, the C group was more accurate than the ML (*p* = .037, 95% CI [-.188, -.005]) but not than the MT group (*p = .*321). Finally, for sadness the C group was more accurate than the ML (*p* = .001, 95% CI [.039, .168]) and the MT group (*p = .*015, 95% CI [.013, .145]).

For the effect of emotion category, the Bonferroni-corrected pairwise comparisons showed that the RT decreased more by the end of the study (i.e. accurate responses became faster) for anger more than fear (*p* < .001, 95% CI [-.650, -.189]), for disgust more than fear (*p* < .001, 95% CI [-.610, -.241]), for joy more than fear (*p* < .001, 95% CI [-.737, -.303]), for sadness more than fear (*p* < .001, 95% CI [-.423, -.154]), for joy more than sadness (*p* = .027, 95% CI [-.447, -.016]), and for joy more than surprise (*p* = .049, 95% CI [-.598, -.001]). That is, RTs for fear were slower than all the other emotion categories except surprise, and RTs for joy were faster than the other emotion categories except for anger and disgust. The main effects found in the results for the group and emotion categories was further explained by the interaction between these two factors. Bonferroni corrected pairwise comparisons , showed that there was no significant difference between groups for anger (*p* ≥ .999), but that the C group was faster than the ML group for disgust (*p* = .040, 95% CI [.014, .751]). For joy, the C (*P* = .008, 95% CI [.110, .871]) and the MT groups (*p* = .004, 95% CI [-.914, -.154]) were faster than the ML group. For sadness, the C group was marginally faster than the ML group (*p* = .053, 95% CI [-.002, .446]), and the MT group was faster than the ML group for surprise (*p = .*020, 95% CI [-.929, -.065]).

**ANOVAs**

We carried out a mixed factorial ANOVA for each emotion separately on the proportion of accurate responses and reaction time (RT) for correct responses with group (music training = MT, music listening = ML and control=C) as between-subjects factor and trial (testing sessions from 1 to 6) and emotion intensity (low, medium and high) as within-subjects factors. T neutral emotion condition was not included because it did not involve changes in intensity. Given the amount of data and results presented here we only reported on the findings for the main effects and interactions involving the group factor.

*Anger*

For accuracy, the analysis revealed no significant main effect of group and no significant interaction effects for group x intensity, group x trial, or group x trial x intensity, *F* ≤ 2.12, *p* ≥ .091 (top panels in Figure S6, S7 and S8). For RT, the analysis showed no significant main effect of group and no interaction effects for group x intensity, group x trial, or group x trial x intensity, *F* ≤ 2.03, *p* ≥ .151 (bottom panels in Figure S6, S7 and S8).


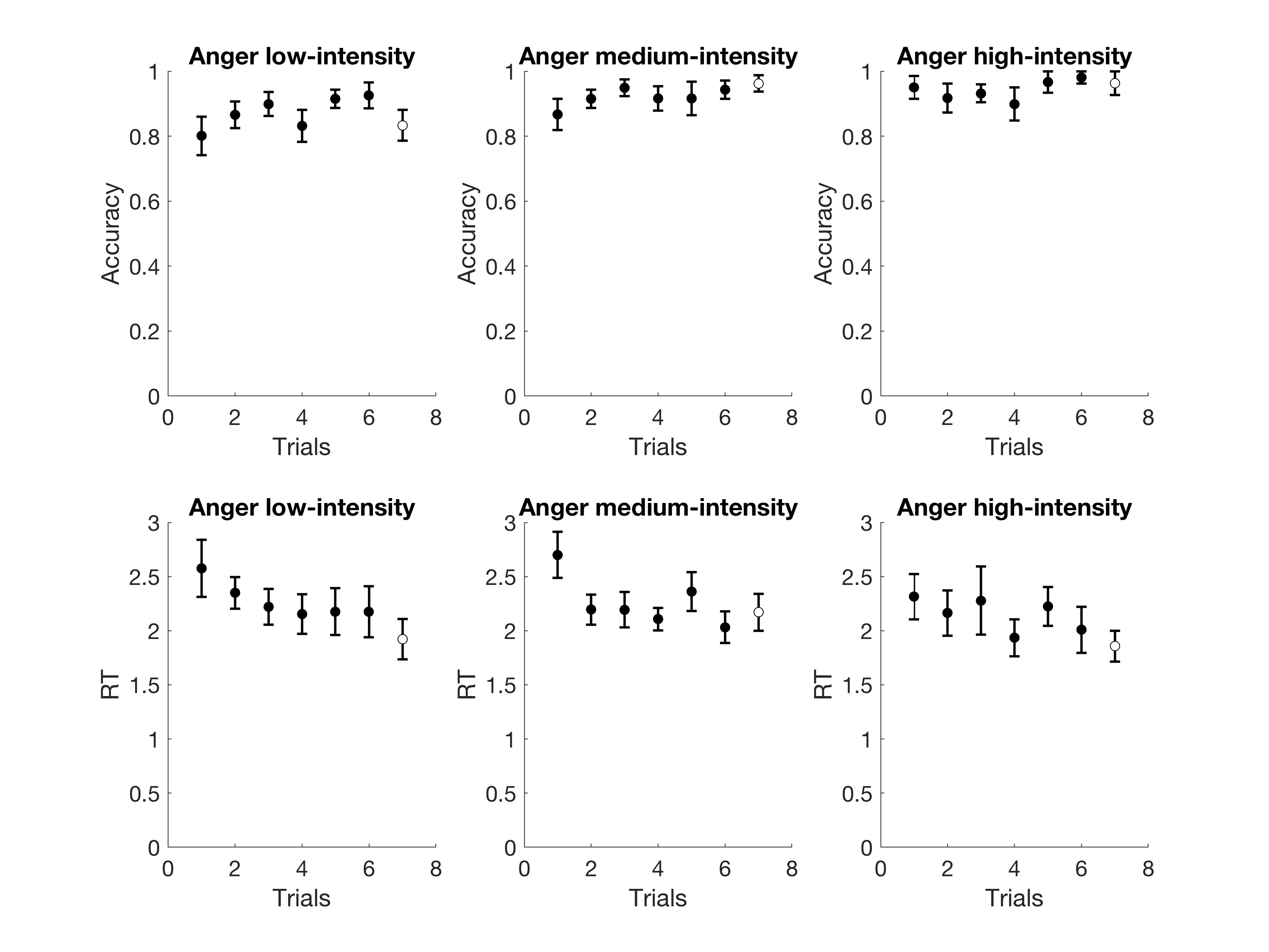


Figure S6. Left panels: Music training group’s performance in emotion recognition for ‘Anger’ at low-intensity as a function of number of trials. Central panels: Music training group’s performance in emotion recognition for ‘Anger’ at medium-intensity as a function of number of trials. Right panels: Music training group’s performance in emotion recognition for ‘Anger’ at high-intensity as a function of number of trials. Top panels represent the average accuracy while bottom panels the average reaction time (RT). The error bars represent the standard errors of the means, and the white circles represent the average mearures at follow-up.


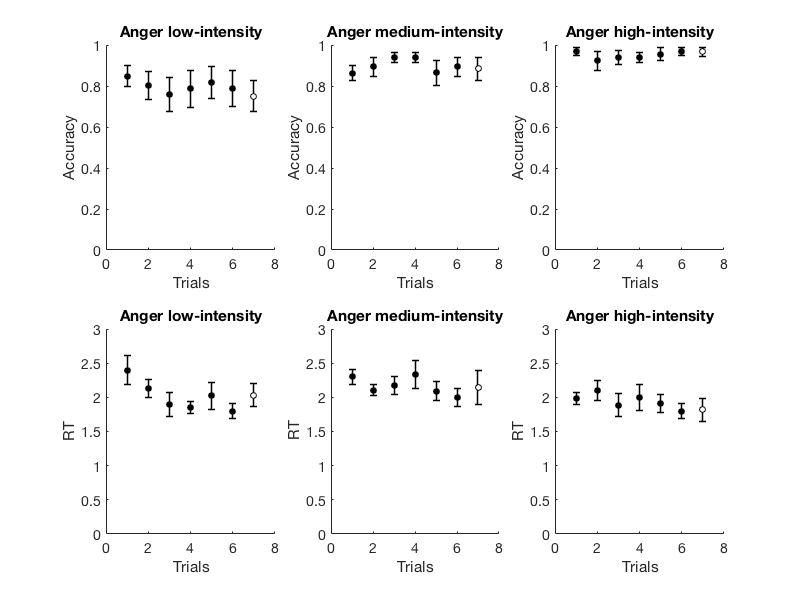


Figure S7. Left panels: Music listening group’s performance in emotion recognition for ‘Anger’ at low- intensity as a function of number of trials. Central panels: Music listening group’s performance in emotion recognition for ‘Anger’ at medium-intensity as a function of number of trials. Right panels: Music listening group’s performance in emotion recognition for ‘Anger’ at high-intensity as a function of number of trials. Top panels represent the average accuracy while bottom panels the average reaction time (RT). The error bars represent the standard errors of the means, and the white circles represent the average mearures at follow-up.


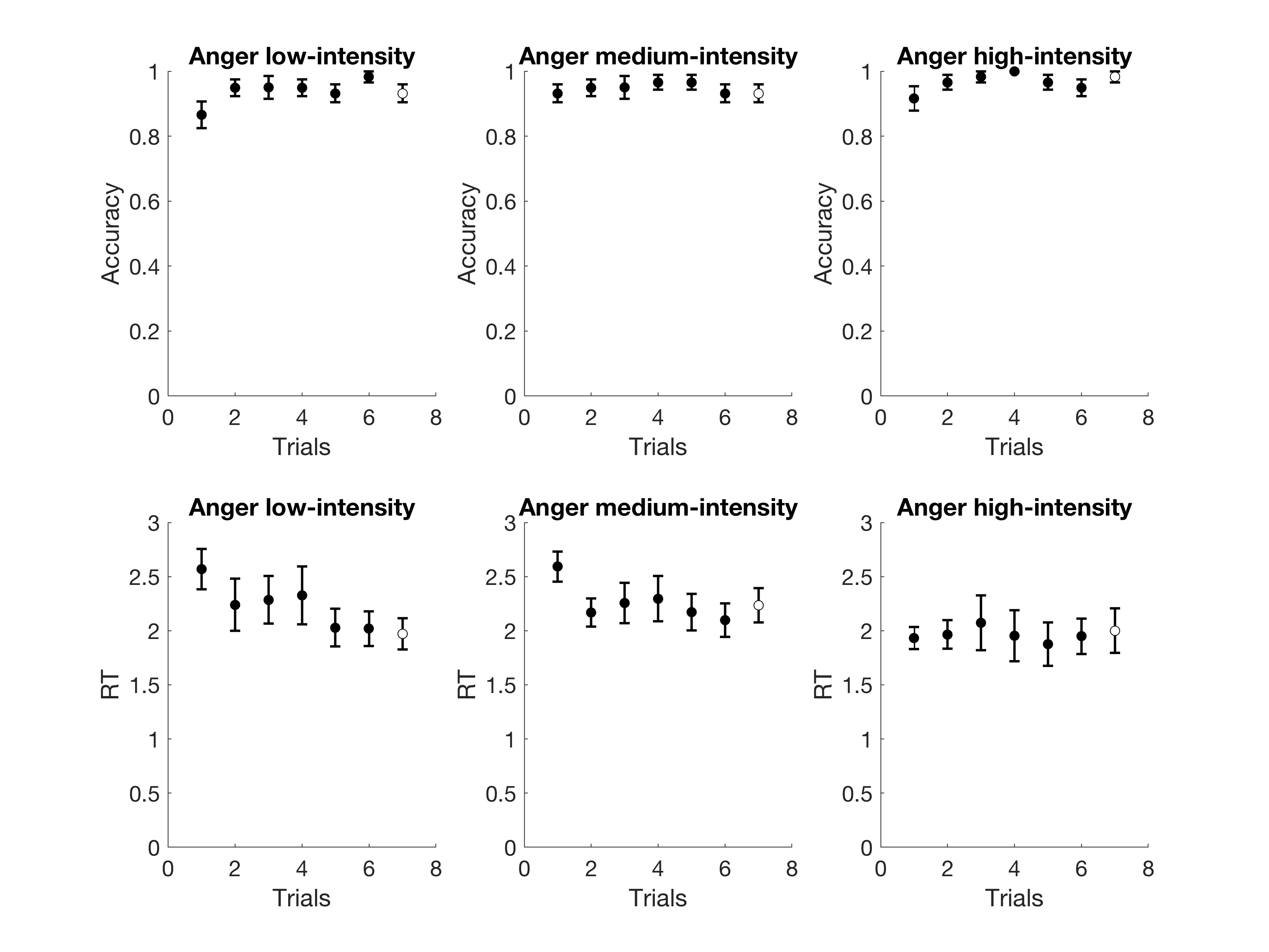


Figure S8. Left panels: Control group’s performance in emotion recognition for ‘Anger’ at low- intensity as a function of number of trials. Central panels: Control group’s performance in emotion recognition for ‘Anger’ at medium-intensity as a function of number of trials. Right panels: Control group’s performance in emotion recognition for ‘Anger’ high-intensity as a function of number of trials. Top panels represent the average accuracy while bottom panels the average reaction time (RT). The error bars represent the standard errors of the means, and the white circles represent the average mearures at follow-up.

*Disgust*

For accuracy, the analysis returned no significant main effect of group and no significant interaction effects for group x intensity, group x trial, or group x trial x intensity, *F* ≤ 1.16, *p* ≥ .324 (top panels in Figure S9, S10 and S11). For RT, the analysis returned no significant main effect of group and no significant interaction effects for group x intensity, group x trial, or group x trial x intensity, *F* ≤ 1.25, *p* ≥ .215 (bottom panels in Figure S9, S10 and S11).


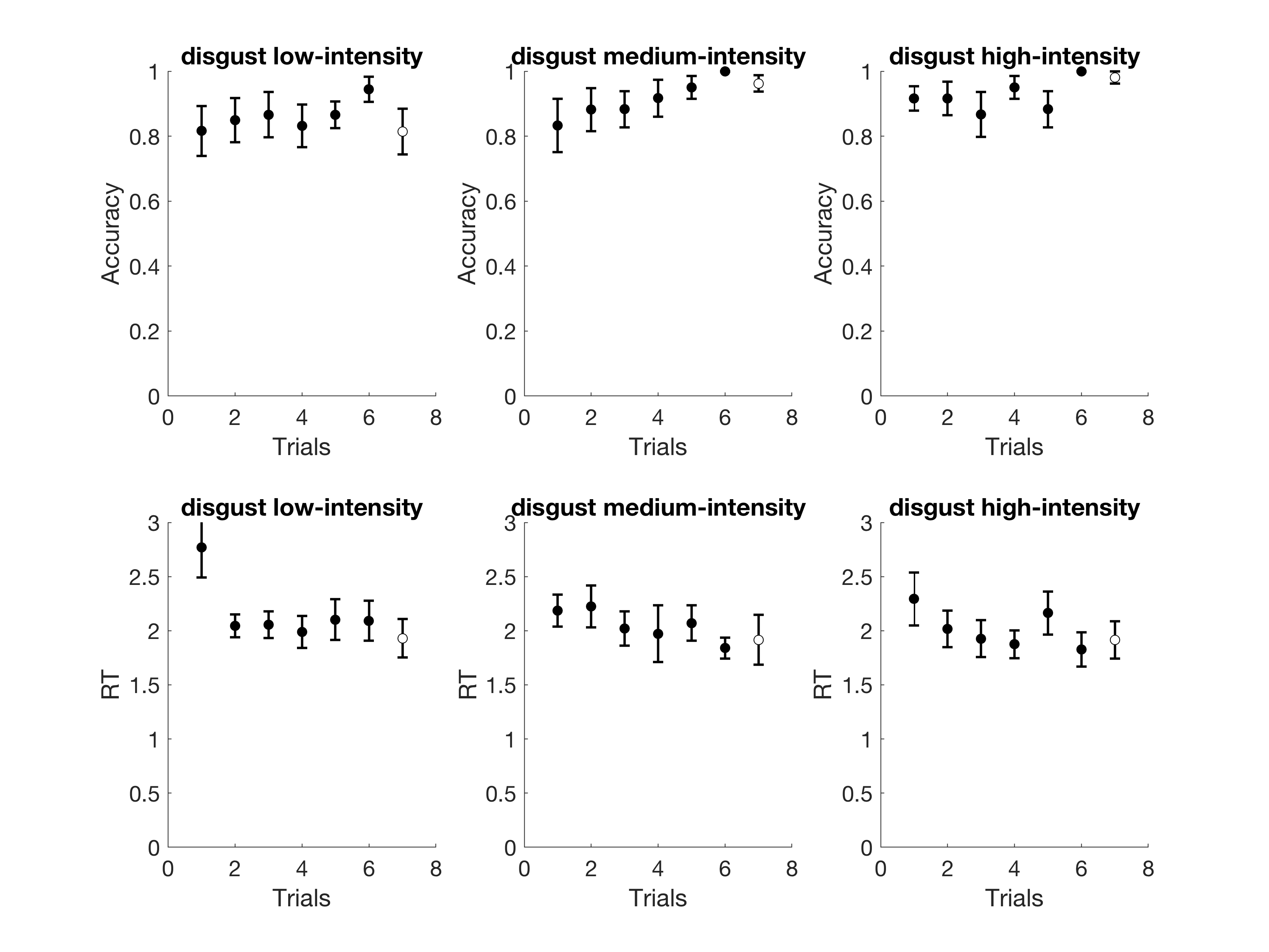


Figure S9. Left panels: Music training group’s performance in emotion recognition for ‘Disgust’ at low- intensity as a function of number of trials. Central panels: Music training group’s performance in emotion recognition for ‘Disgust’ at medium-intensity as a function of number of trials. Right panels: Music training group’s performance in emotion recognition for ‘Disgust’ at high-intensity as a function of number of trials. Top panels represent the average accuracy while bottom panels the average reaction time (RT). The error bars represent the standard errors of the means, and the white circles represent the average mearures at follow-up.


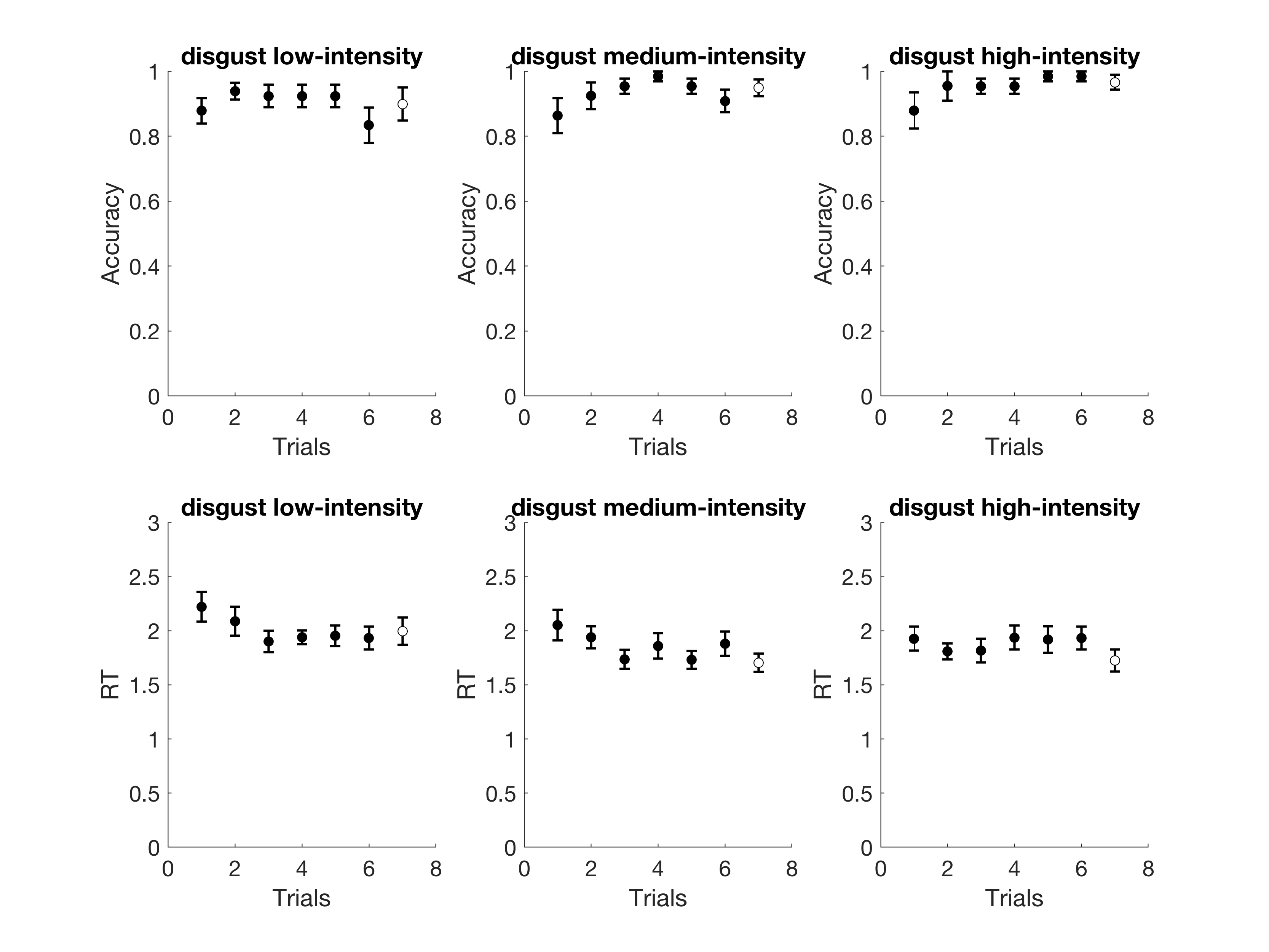


Figure S10. Left panels: Music listening group’s performance in emotion recognition for ‘Disgust’ at low- intensity as a function of number of trials. Central panels: Music listening group’s performance in emotion recognition for ‘Disgust’ at medium-intensity as a function of number of trials. Right panels: Music listening group’s performance in emotion recognition for ‘Disgust’ at high-intensity as a function of number of trials. Top panels represent the average accuracy while bottom panels the average reaction time (RT). The error bars represent the standard errors of the means, and the white circles represent the average mearures at follow-up.


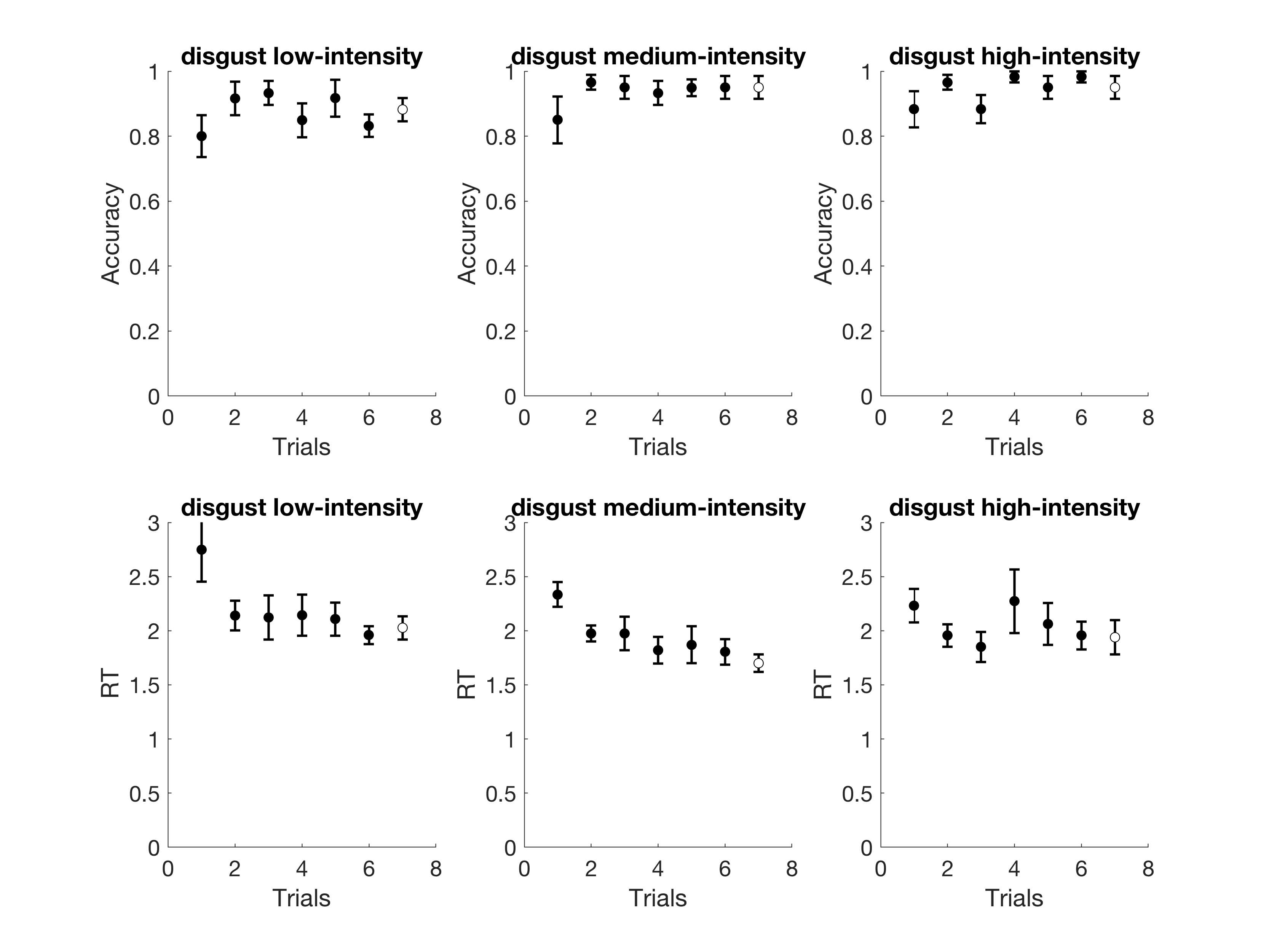


Figure S11. Left panels: Control group’s performance in emotion recognition for ‘Disgust’ at low- intensity as a function of number of trials. Central panels: Control group’s performance in emotion recognition for ‘Disgust’ at medium-intensity as a function of number of trials. Right panels: Control group’s performance in emotion recognition for ‘Disgust’ at high-intensity as a function of number of trials. Top panels represent the average accuracy while bottom panels the average reaction time (RT). The error bars represent the standard errors of the means, and the white circles represent the average mearures at follow-up.

*Fear*

For accuracy, the analysis showed no significant main effect of group and no significant interaction effects for group x intensity, group x trial, or group x trial x intensity, *F* ≤ 1.30, *p* ≥ .176 (top panels in Figure S12, S13 and S14). For RT, the analysis revealed no significant main effect of group and no significant interaction effects for group x intensity, group x trial, or group x trial x intensity, *F* ≤ 1.58, *p* ≥ .195 (bottom panels in Figure S12, S13 and S14).


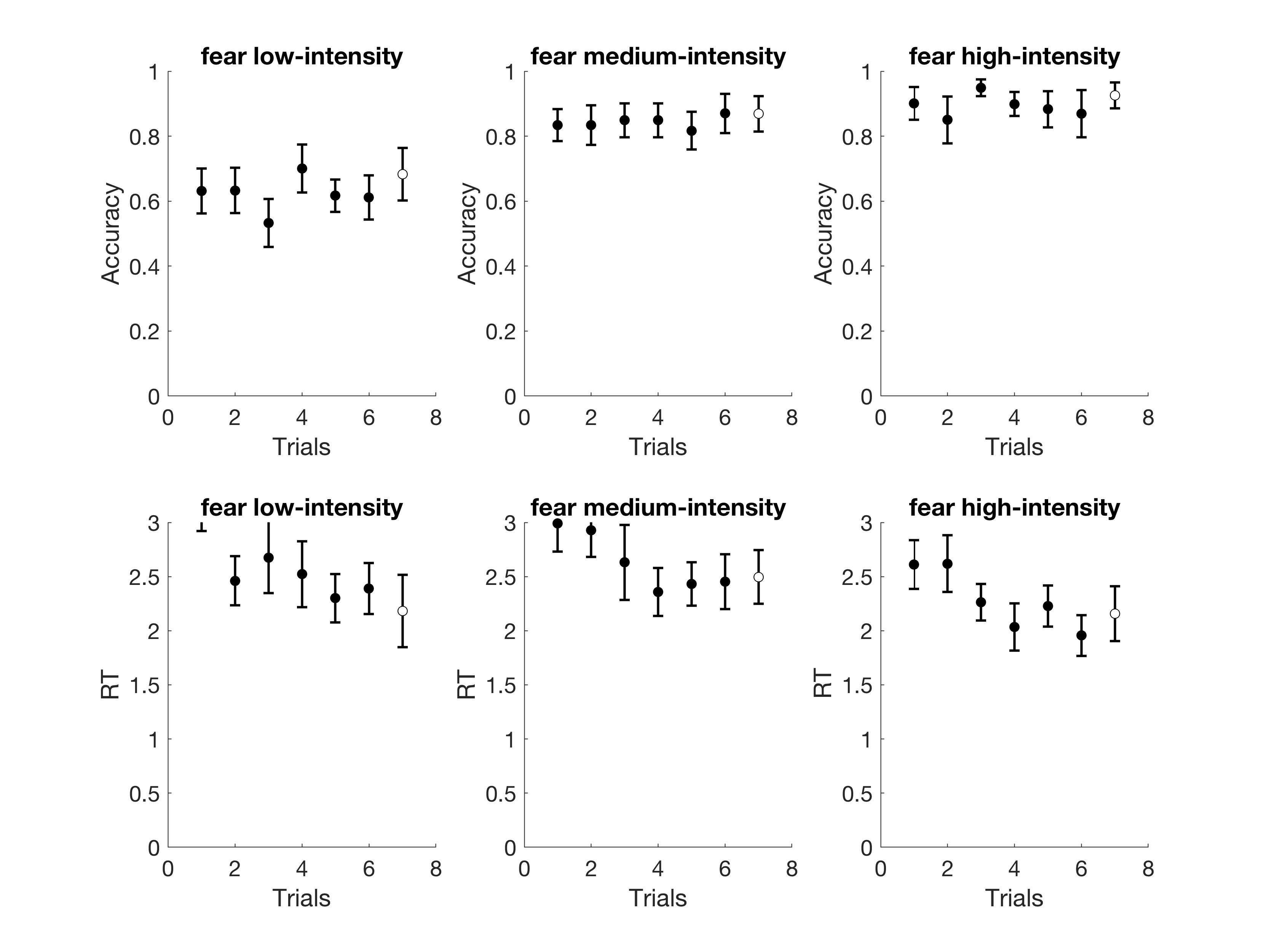


Figure S12. Left panels: Music training group’s performance in emotion recognition for ‘Fear’ at low- intensity as a function of number of trials. Central panels: Music training group’s performance in emotion recognition for ‘Fear’ at medium-intensity as a function of number of trials. Right panels: Music training group’s performance in emotion recognition for ‘Fear’ at high-intensity as a function of number of trials. Top panels represent the average accuracy while bottom panels the average reaction time (RT). The error bars represent the standard errors of the means, and the white circles represent the average mearures at follow-up.


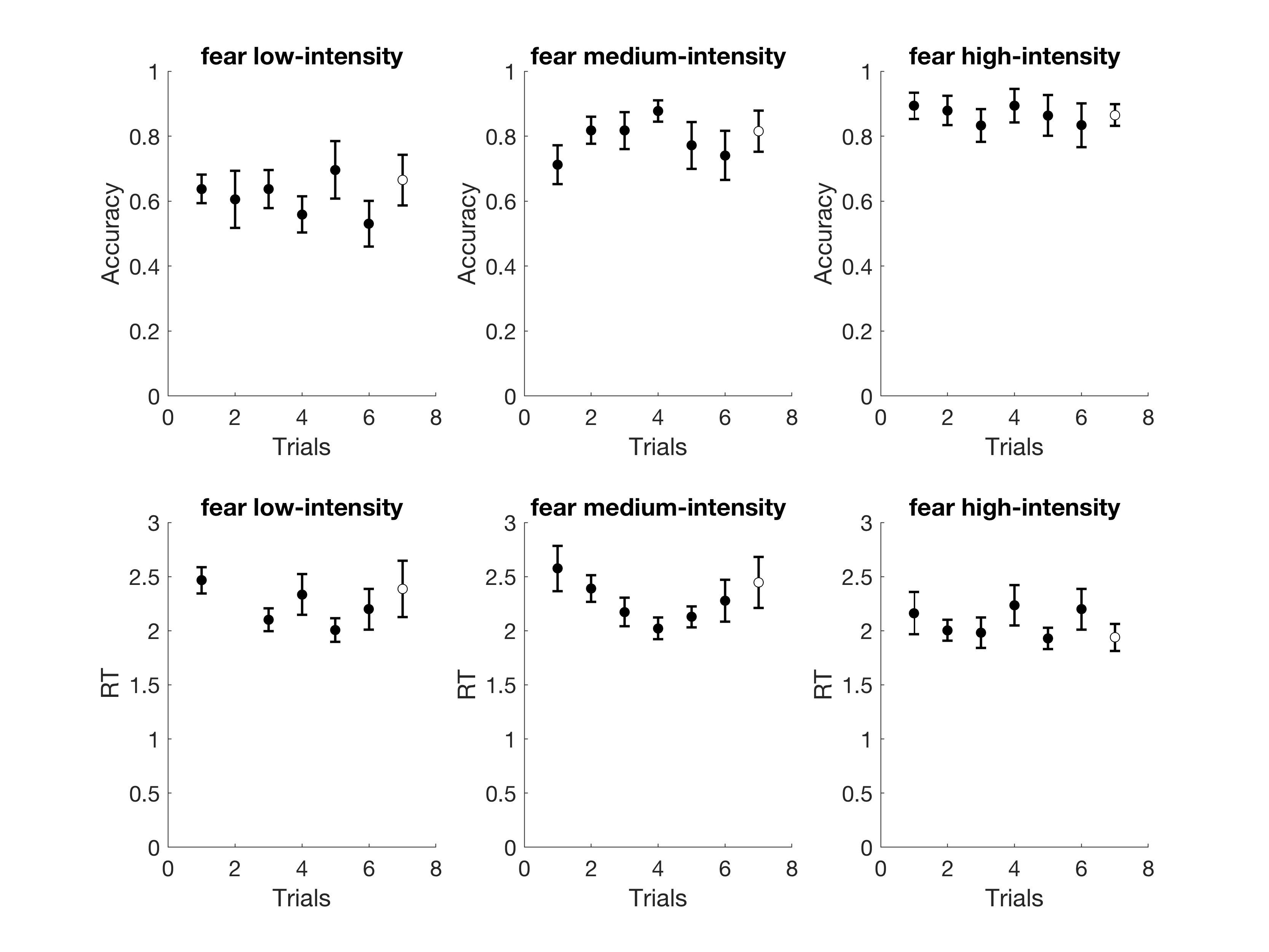


Figure S13. Left panels: Music listening group’s performance in emotion recognition for ‘Fear’ at low- intensity as a function of number of trials. Central panels: Music listening group’s performance in emotion recognition for ‘Fear’ at medium-intensity as a function of number of trials. Right panels: Music listening group’s performance in emotion recognition for ‘Fear’ at high-intensity as a function of number of trials. Top panels represent the average accuracy while bottom panels the average reaction time (RT). The error bars represent the standard errors of the means, and the white circles represent the average mearures at follow-up.


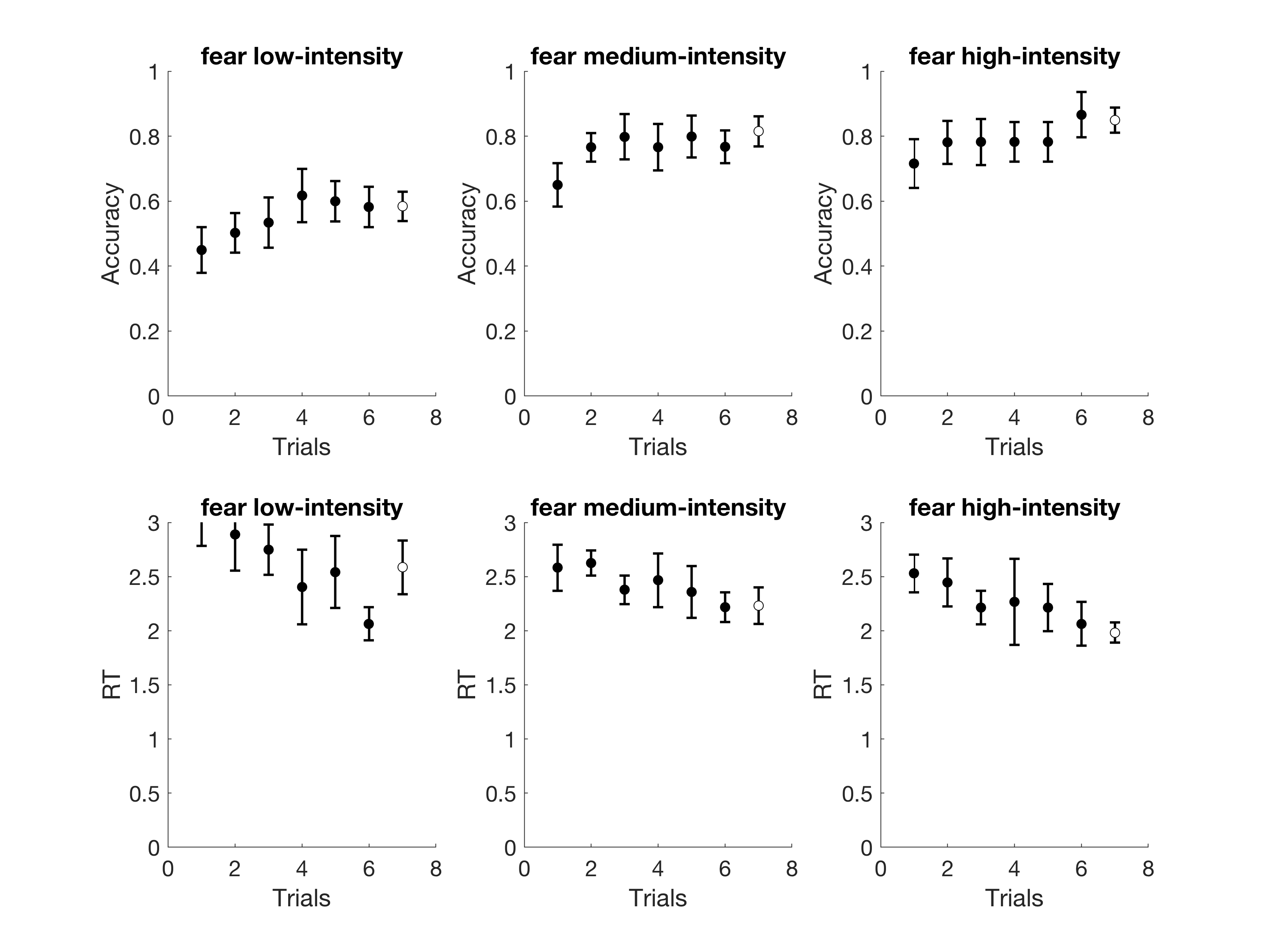


Figure S14. Left panels: Control group’s performance in emotion recognition for ‘Fear’ at low-intensity as a function of number of trials. Central panels: Control group’s performance in emotion recognition for ‘Fear’ at medium-intensity as a function of number of trials. Right panels: Control group’s performance in emotion recognition for ‘Fear’ at high-intensity as a function of number of trials. Top panels represent the average accuracy while bottom panels the average reaction time (RT). The error bars represent the standard errors of the means, and the white circles represent the average mearures at follow-up.

*Joy*

For accuracy, the analysis showed no significant main effect of group and no significant interaction of intensity x group. However, significant interaction effects were found for trial x group, *F*(10, 135) = 2.42, *p* = .011, and f trial x intensity x group, *F*(20, 270) = 1.99, *p* = .008. Bonferroni corrected pairwise comparisons showed that the significant effect of trial x group was due to the increasing accuracy across trials for the control group, but not for the music training or listening group, in recognising joyafter the first trial (1^st^ vs 2^nd^ trial: *p* = .048; 1^st^ vs 3^rd^ trial: *p* = .014; 1^st^ vs 4^th^ trial: *p* =.013; 1^st^ vs 5^th^ trial: *p* =.005; 1^st^ vs 6^th^ trial: *p* = .023). The significant trial x intensity x group interaction was once again due to the C group, but not the other groups, significantly improving in recognising joy after the first trial, specifically for the low emotion intensity (1^st^ vs 2^nd^ trial: *p* = .036; 1^st^ vs 3^rd^ trial: *p* = .026; 1^st^ vs 4^th^ trial: *p* =.004; 1^st^ vs 5^th^ trial: *p* =.001; 1^st^ vs 6^th^ trial: *p* = .009). See top panels in Figure S15, S16 and S17. For RT, the analysis returned a significant trial x intensity x group interaction effect, *F*(20, 270) = 1.72, *p* = .030. No significant main effect of group or interaction effects of trial x group or intensity x group effect were found. Bonferroni corrected pairwise comparisons showed that the significant interaction effect of trial x intensity x group was due to the RTs of the MT group, but not the ML or C group, significantly decreasing after the second trial for the low emotion intensity (1^st^ vs 2^nd^ trial: *p* = .117; 1^st^ vs 3^rd^ trial: *p* = .015; 1^st^ vs 4^th^ trial: *p* =.010; 1^st^ vs 5^th^ trial: *p* =.007; 1^st^ vs 6^th^ trial: *p* < .001). See bottom panels in Figure S15, S16 and S17.


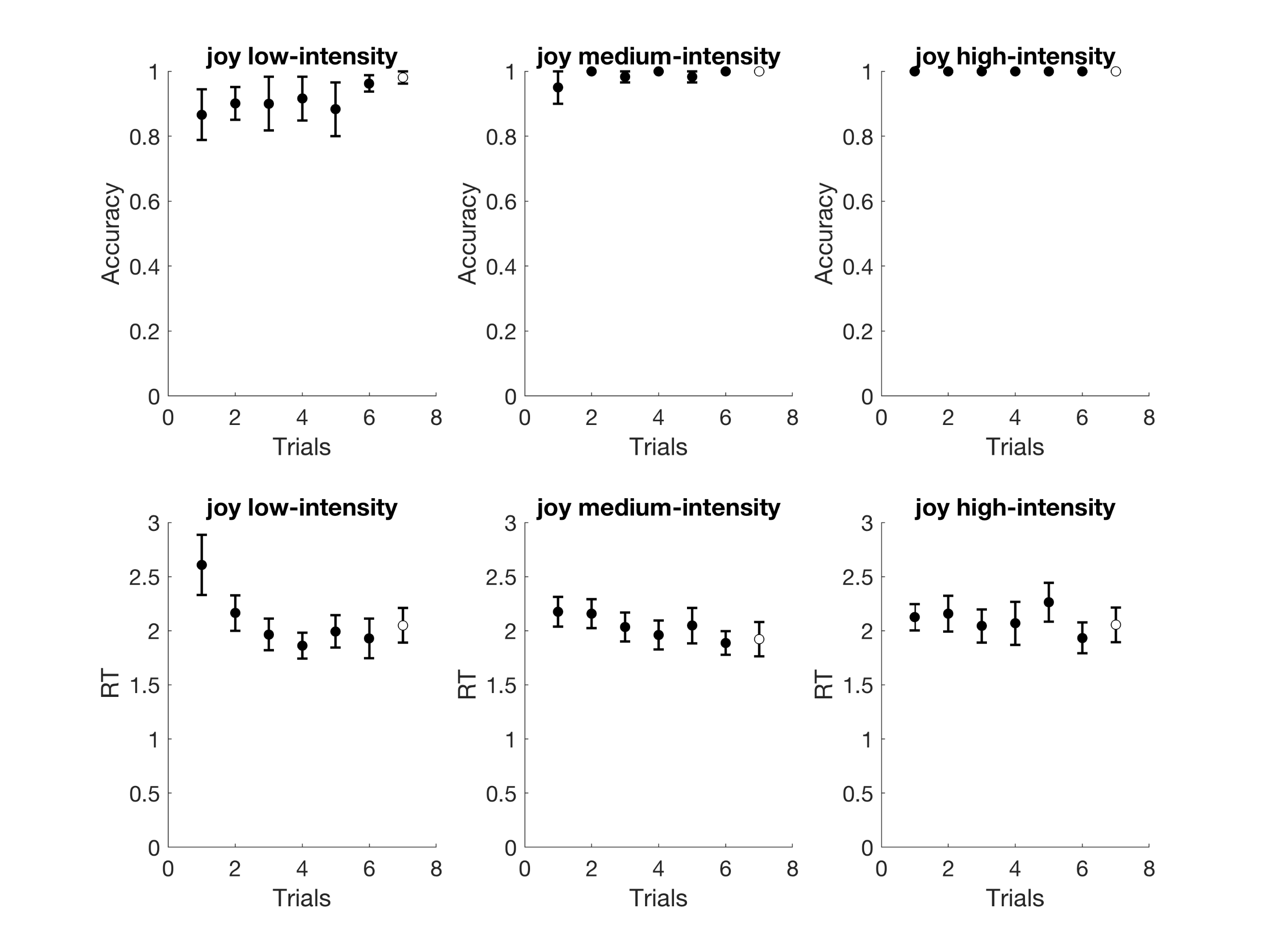


Figure S15. Left panels: Music training group’s performance in emotion recognition for ‘Joy’ at low- intensity as a function of number of trials. Central panels: Music training group’s performance in emotion recognition for ‘Joy’ at medium-intensity as a function of number of trials. Right panels: Music training group’s performance in emotion recognition for ‘Joy’ at high-intensity as a function of number of trials. Top panels represent the average accuracy while bottom panels the average reaction time (RT). The error bars represent the standard errors of the means, and the white circles represent the average mearures at follow-up.


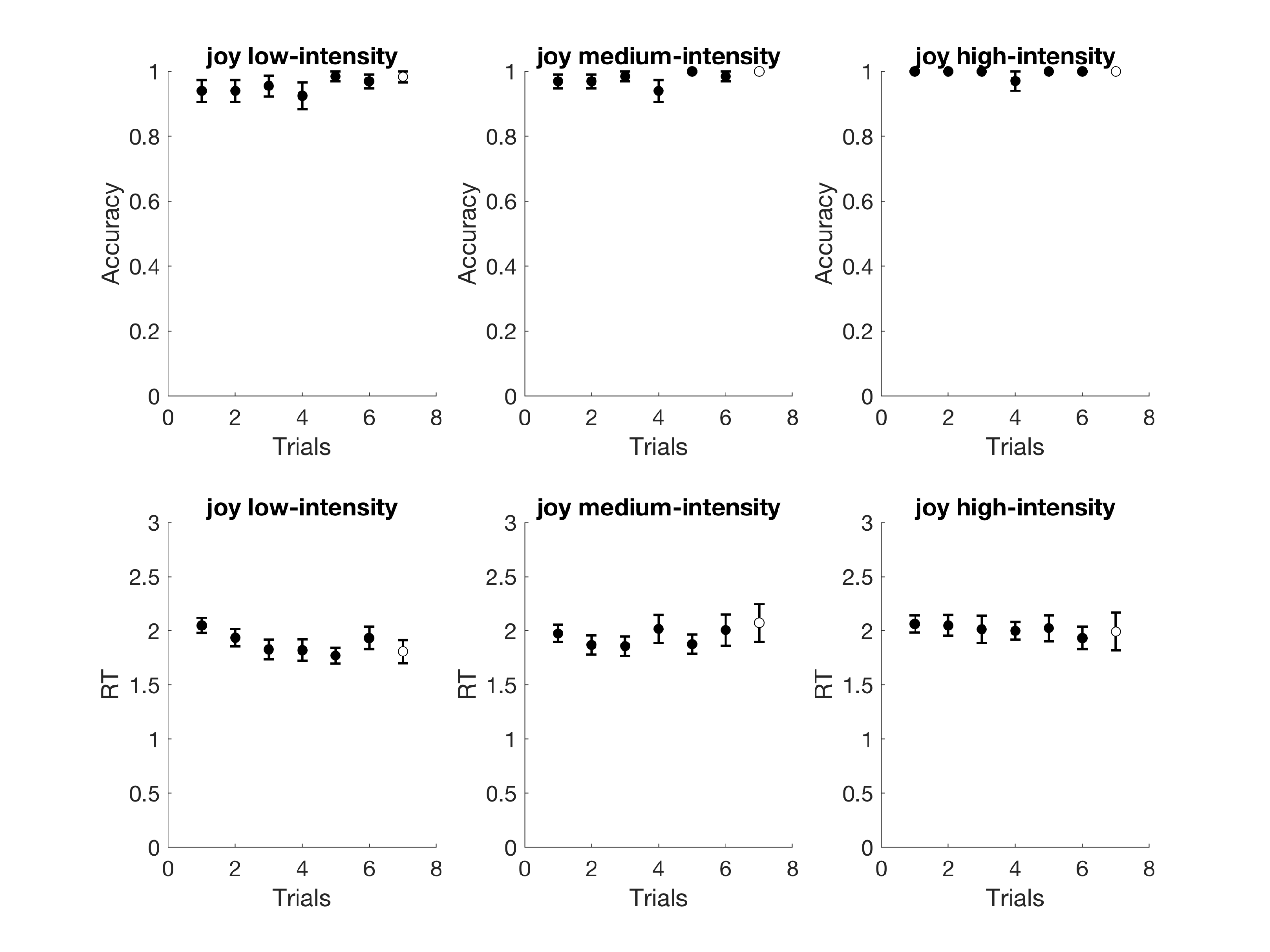


Figure S16. Left panels: Music listening group’s performance in emotion recognition for ‘Joy’ at low- intensity as a function of number of trials. Central panels: Music listening group’s performance in emotion recognition for ‘Joy’ at medium-intensity as a function of number of trials. Right panels: Music listening group’s performance in emotion recognition for ‘Joy’ at high-intensity as a function of number of trials. Top panels represent the average accuracy while bottom panels the average reaction time (RT). The error bars represent the standard errors of the means, and the white circles represent the average mearures at follow-up.


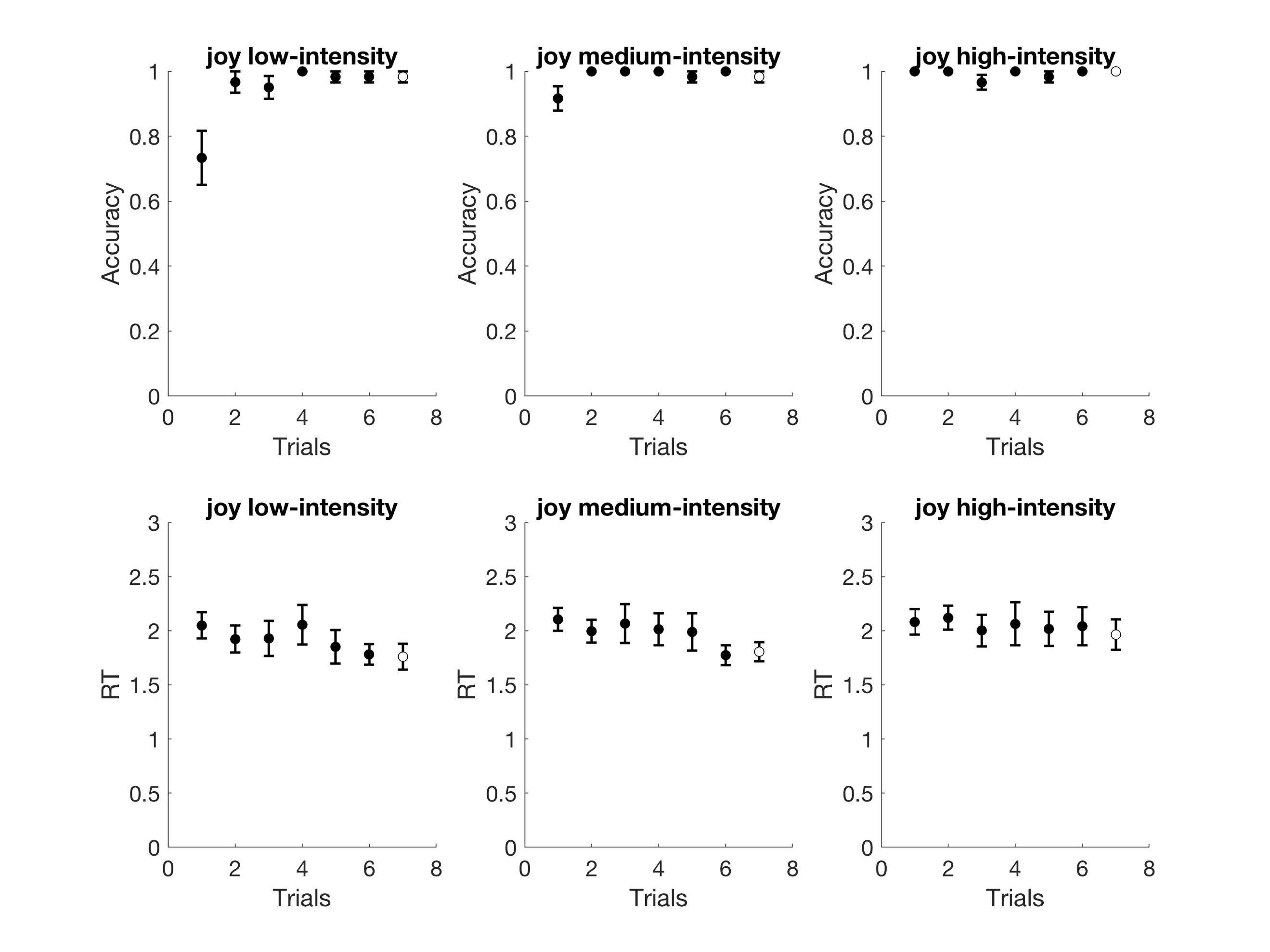


Figure S17. Left panels: Control group’s performance in emotion recognition for ‘Joy’ at low-intensity as a function of number of trials. Central panels: Control group’s performance in emotion recognition for ‘Joy’ at medium-intensity as a function of number of trials. Right panels: Control group’s performance in emotion recognition for ‘Joy’ at high-intensity as a function of number of trials. Top panels represent the average accuracy while bottom panels the average reaction time (RT). The error bars represent the standard errors of the means, and the white circles represent the average mearures at follow-up.

*Sadness*

For accuracy, the analysis showed a significant trial x group interaction effect , *F*(10, 135) = 3.15, *p* = .001. No significant main effect of group or interaction effect of intensity x group or trial x intensity x group was found. Bonferroni corrected pairwise comparisons showed that the significant trial x group interaction effect was due to the C group being significantly less accurate at trial 1 than the other groups (C vs MT: *p* = .036; C vs ML: *p* = .028). See top panels in Figure S18, S19 and S20. For RT, the analysis returned no significant main effect of group or interactions of group x intensity, group x trial, or group x trial x intensity, *F* ≤ 1.45, *p* ≥ .100 (bottom panels in Figure S18, S19 and S20).


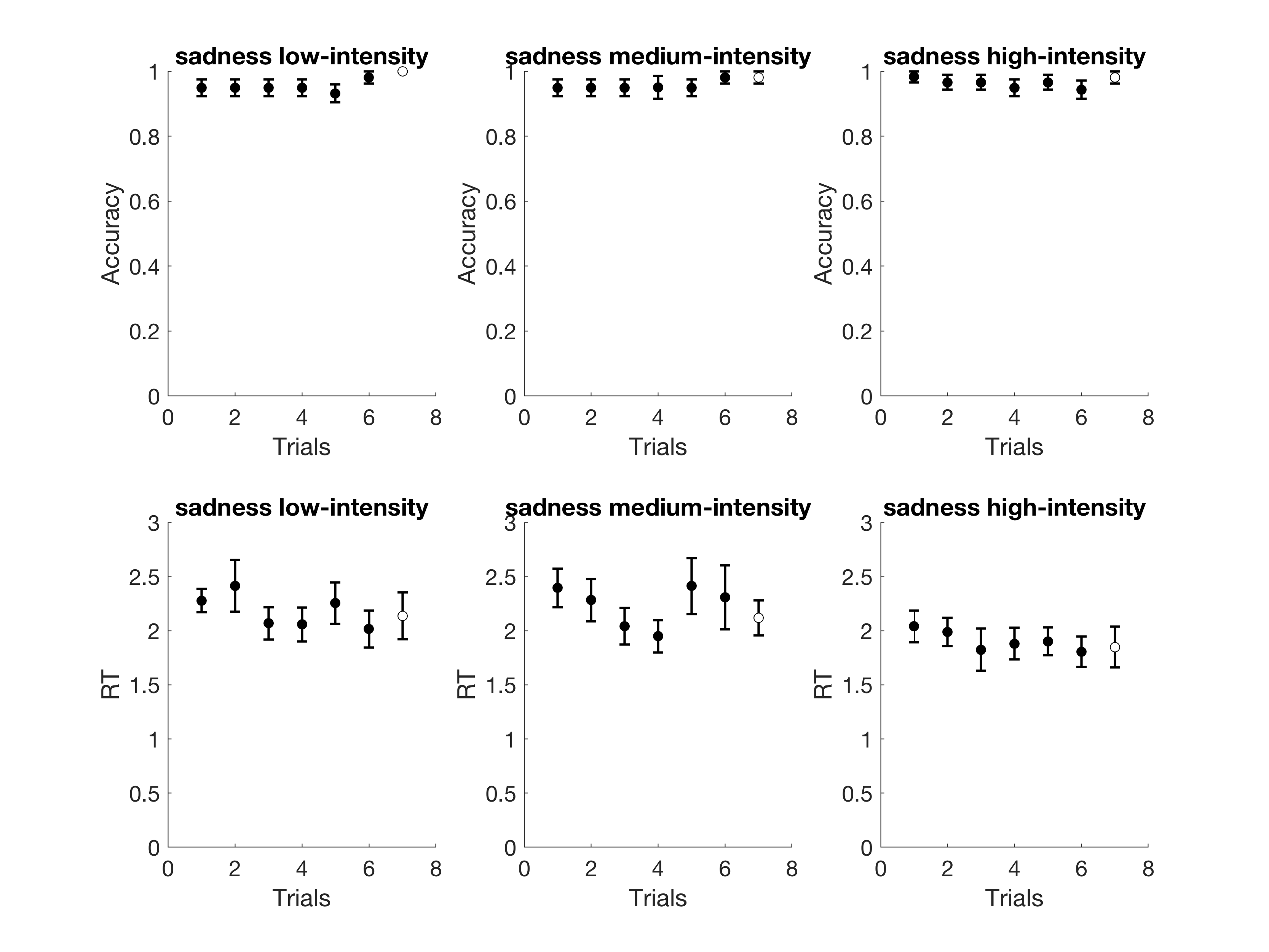


Figure S18. Left panels: Music training group’s performance in emotion recognition for ‘Sadness’ at low-intensity as a function of number of trials. Central panels: Music training group’s performance in emotion recognition for ‘Sadness’ at medium-intensity as a function of number of trials. Right panels: Music training group’s performance in emotion recognition for ‘Sadness’ at high-intensity as a function of number of trials. Top panels represent the average accuracy while bottom panels the average reaction time (RT). The error bars represent the standard errors of the means, and the white circles represent the average mearures at follow-up.


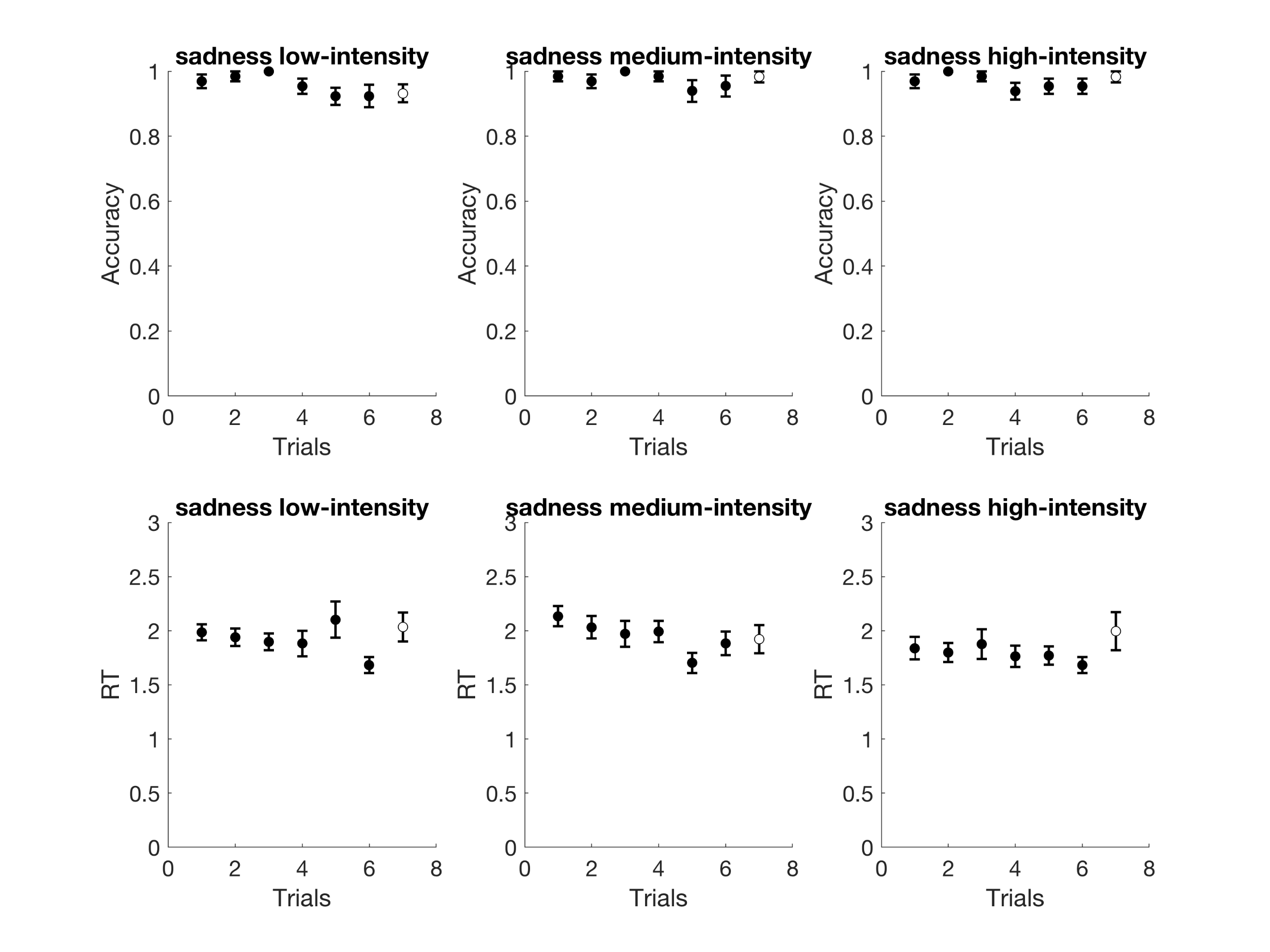


Figure S19. Left panels: Music listening group’s performance in emotion recognition for ‘Sadness’ at low-intensity as a function of number of trials. Central panels: Music listening group’s performance in emotion recognition for ‘Sadness’ at medium-intensity as a function of number of trials. Right panels: Music listening group’s performance in emotion recognition for ‘Sadness’ at high-intensity as a function of number of trials. Top panels represent the average accuracy while bottom panels the average reaction time (RT). The error bars represent the standard errors of the means, and the white circles represent the average mearures at follow-up.


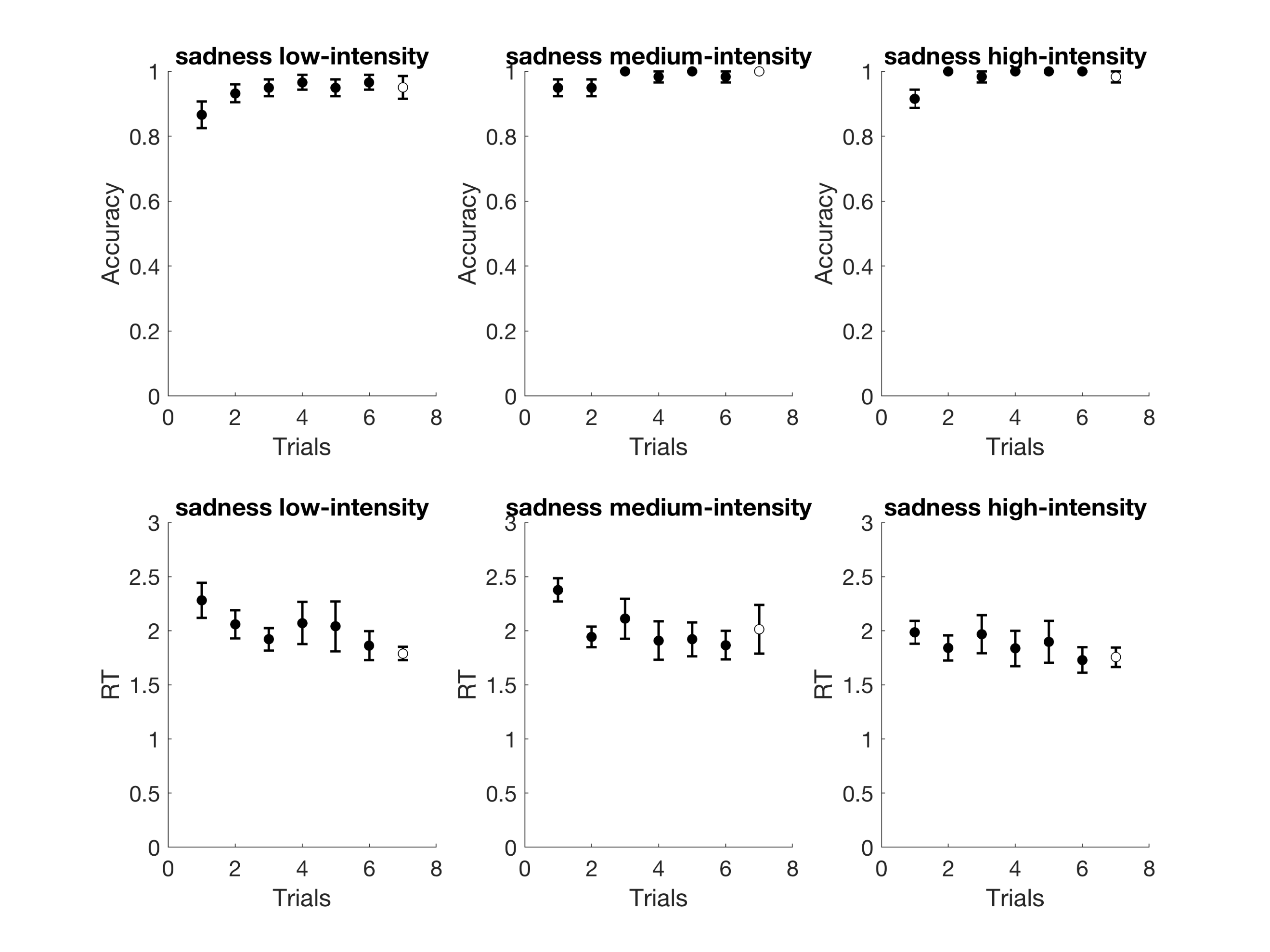


Figure S20. Left panels: Control group’s performance in emotion recognition for ‘Sadness’ at low- intensity as a function of number of trials. Central panels: Control group’s performance in emotion recognition for ‘Sadness’ at medium-intensity as a function of number of trials. Right panels: Control group’s performance in emotion recognition for ‘Sadness’ at high-intensity as a function of number of trials. Top panels represent the average accuracy while bottom panels the average reaction time (RT). The error bars represent the standard errors of the means, and the white circles represent the average mearures at follow-up.

*Surprise*

For accuracy, the analysis returned a significant interaction effect of trial x intensity x group, *F*(20, 270) = 1.70, *p* = .034. No significant main effect of group or interaction effect of intensity x group or trial x group was found. Bonferroni corrected pairwise comparisons showed that the significant interaction effect of trial x intensity x group was due to only the ML group differing significantly in accuracy at trial 5 and 6 for the medium vs high emotion intensity (*p* = .040), and for the low vs medium emotion intensity respectively (*p* = .006). See top panels in Figure S21, S22and S23. For RT, the analysis returned no significant main effect of group or interaction effect of group x intensity, group x trial, or group x trial x intensity, *F* ≤ 1.65, *p* ≥ .210 (bottom panels in Figure S21, S22and S23).


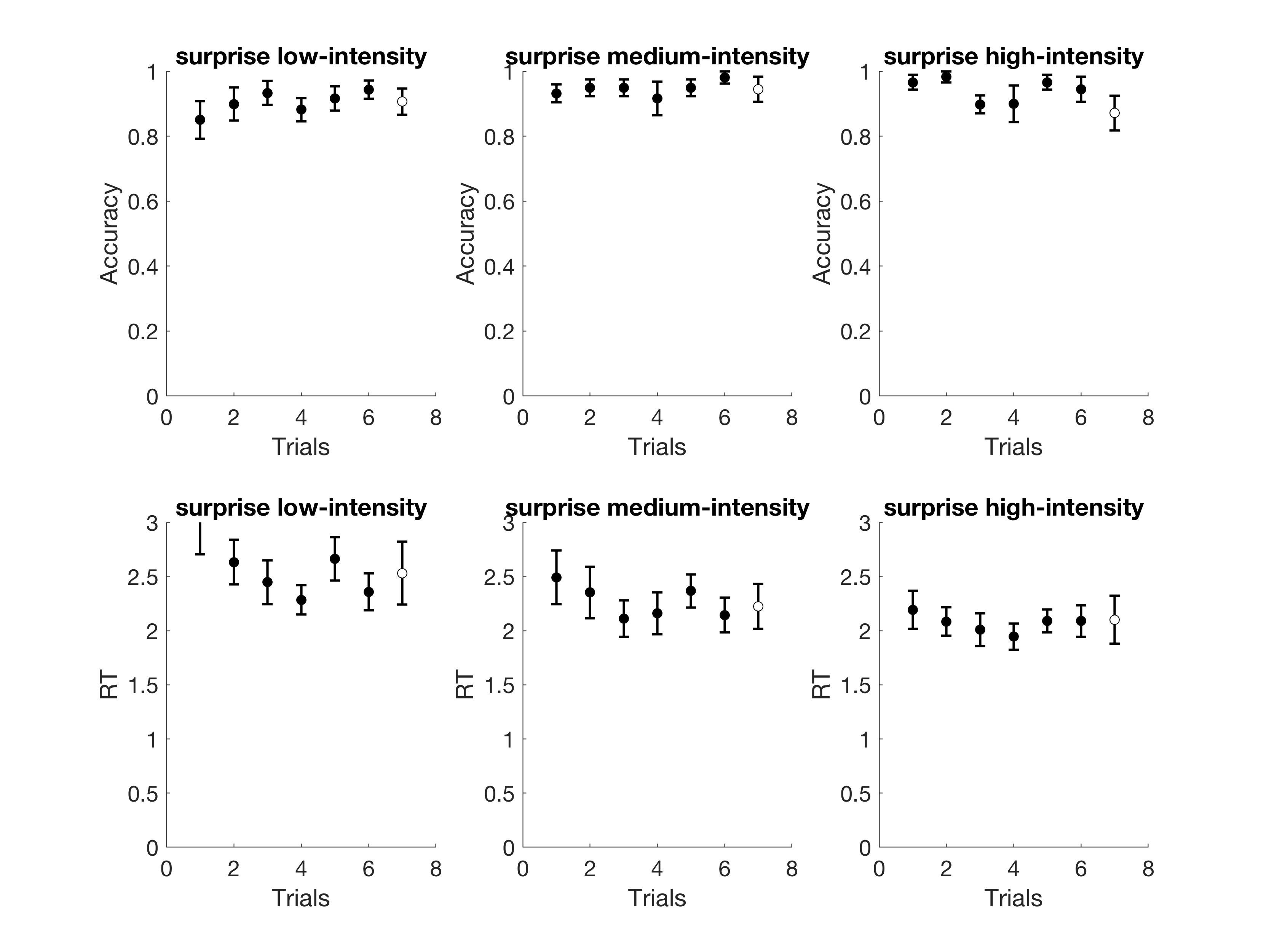


Figure S21. Left panels: Music training group’s performance in emotion recognition for ‘Surprise’ at low-intensity as a function of number of trials. Central panels: Music training group’s performance in emotion recognition for ‘Surprise’ at medium-intensity as a function of number of trials. Right panels: Music training group’s performance in emotion recognition for ‘Surprise’ at high-intensity as a function of number of trials. Top panels represent the average accuracy while bottom panels the average reaction time (RT). The error bars represent the standard errors of the means, and the white circles represent the average mearures at follow-up.


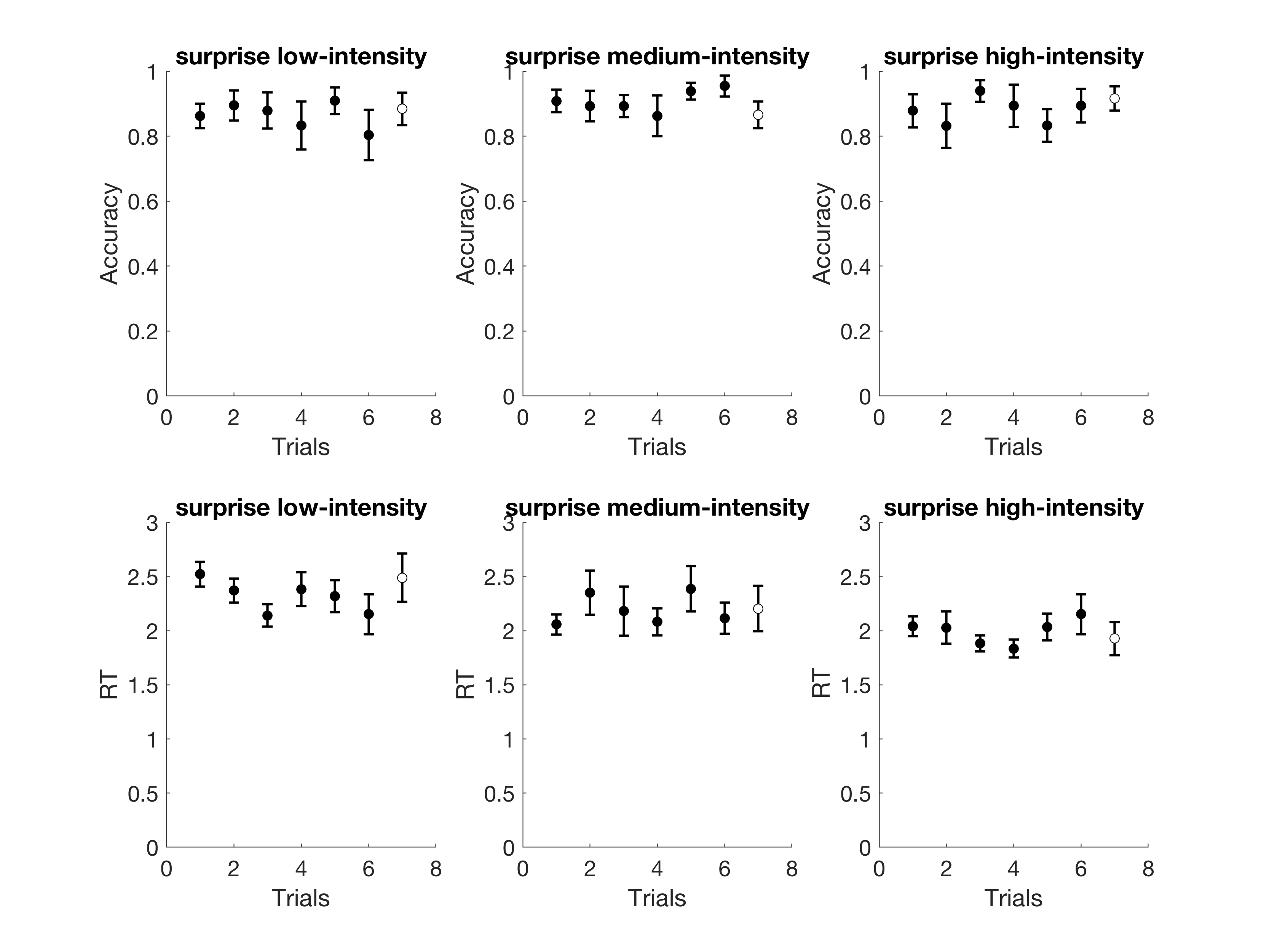


Figure S22. Left panels: Music listening group’s performance in emotion recognition for ‘Surprise’ at low-intensity as a function of number of trials. Central panels: Music listening group’s performance in emotion recognition for ‘Surprise’ at medium-intensity as a function of number of trials. Right panels: Music listening group’s performance in emotion recognition for ‘Surprise’ at high-intensity as a function of number of trials. Top panels represent the average accuracy while bottom panels the average reaction time (RT). The error bars represent the standard errors of the means, and the white circles represent the average mearures at follow-up.


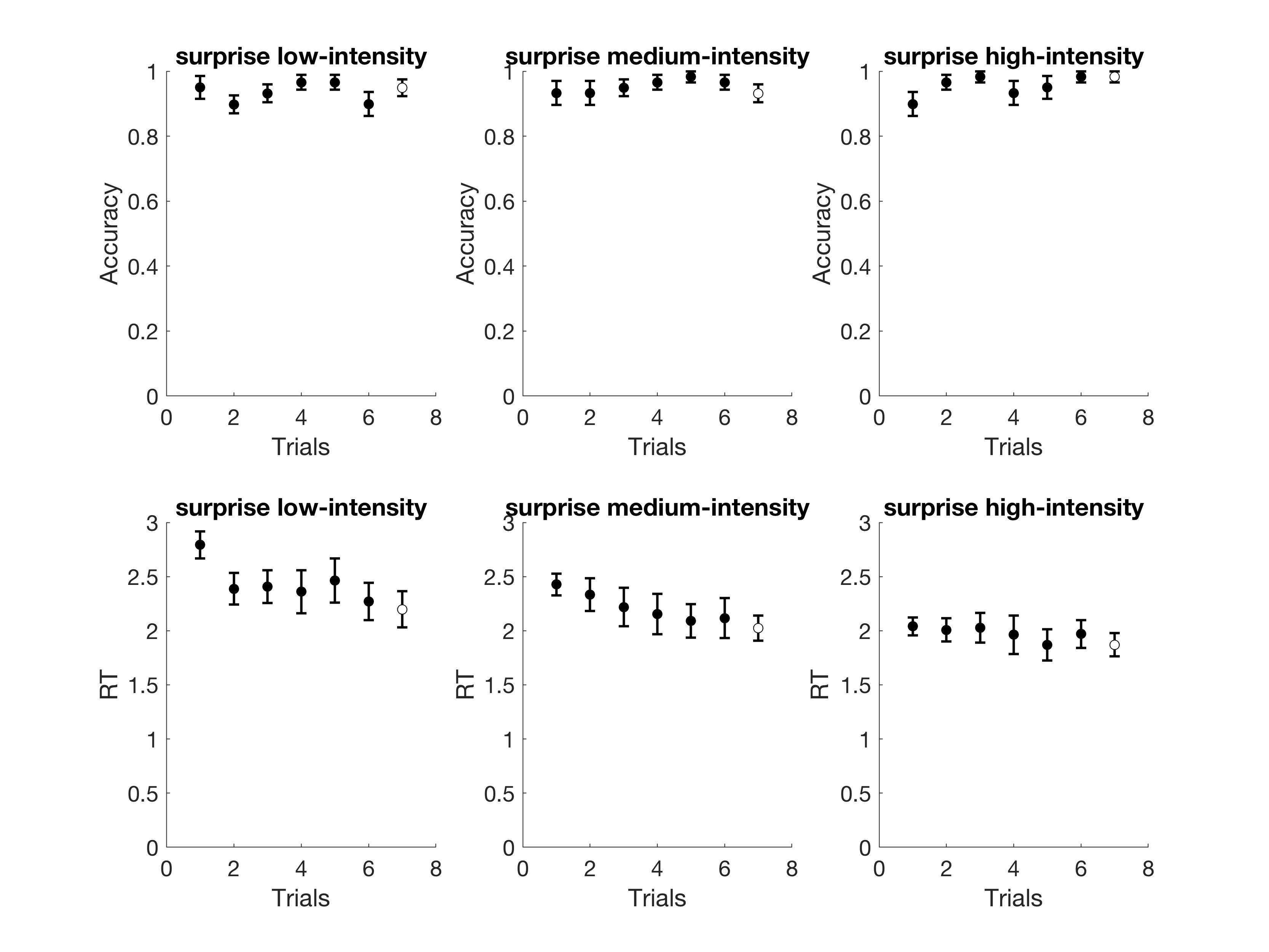


Figure S23. Left panels: Control group’s performance in emotion recognition for ‘Surprise’ at low- intensity as a function of number of trials. Central panels: Control group’s performance in emotion recognition for ‘Surprise’ at medium-intensity as a function of number of trials. Right panels: Control group’s performance in emotion recognition for ‘Surprise’ at high-intensity as a function of number of trials. Top panels represent the average accuracy while bottom panels the average reaction time (RT). The error bars represent the standard errors of the means, and the white circles represent the average mearures at follow-up.

*Neutral*

For accuracy, the analysis revealed no significant main effect of group or group x trial interaction effect, *F* ≤ 0.95, *p* ≥ .486 (top panels in Figure S24, S25 and S26). For RT, the analysis showed no significant main effect of group or interaction effect for group x intensity, group x trial, or group x trial x intensity, *F* ≤ 0.42, *p* ≥ .664 (bottom panels in Figure S24, S25 and S26).


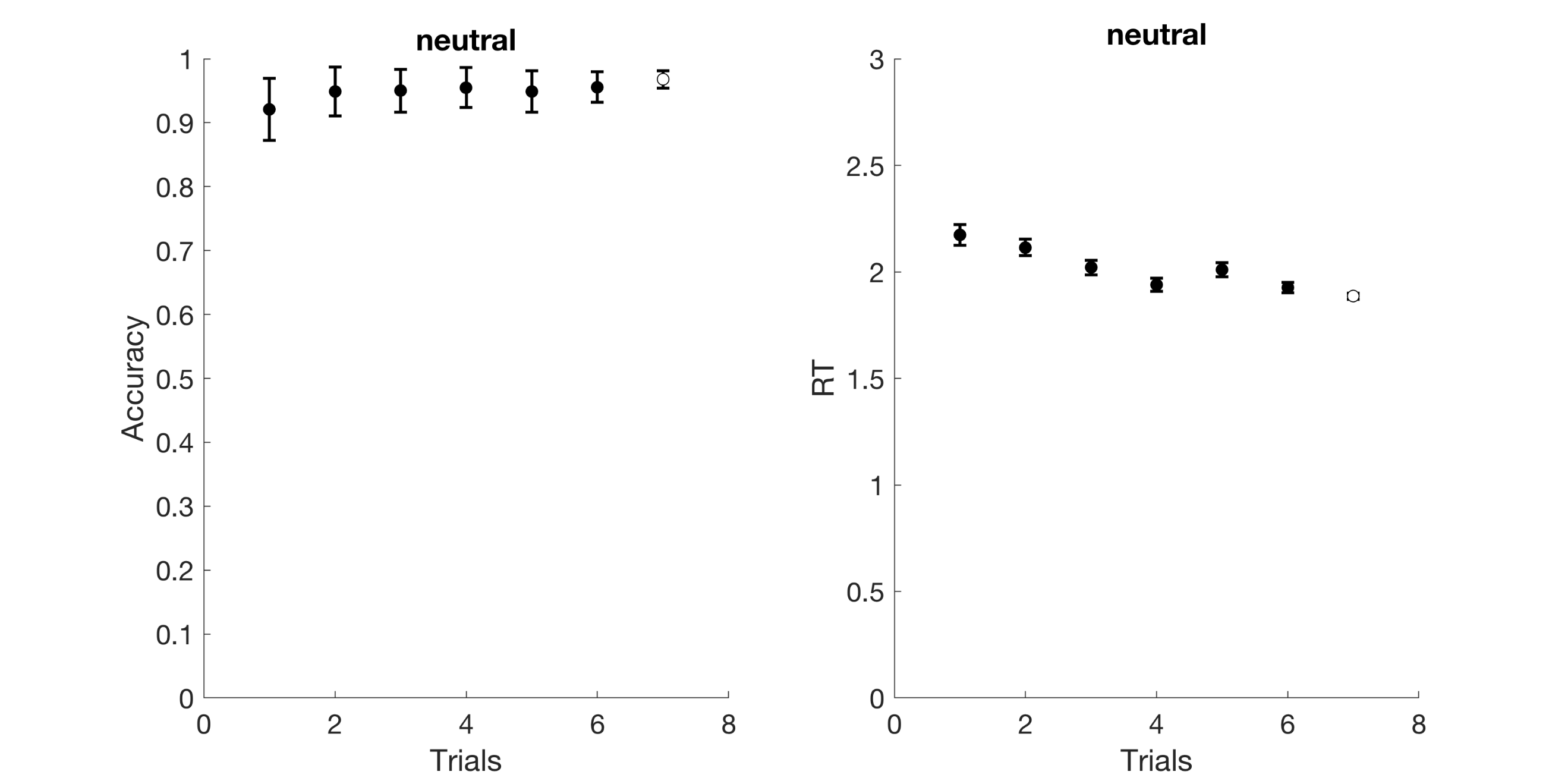


Figure S24. Left panels: Music training group’s performance in emotion recognition for ‘Neutral’ as a function of number of trials for the average accuracy. Right panels: Music training group’s performance in emotion recognition for ‘Neutral’ as a function of number of trials for the average reaction time (RT). The error bars represent the standard errors of the means, and the white circle the follow-up measurement of ‘Neutral’ recognition accuracy and RT. Note: neutral had no variations in intensity.


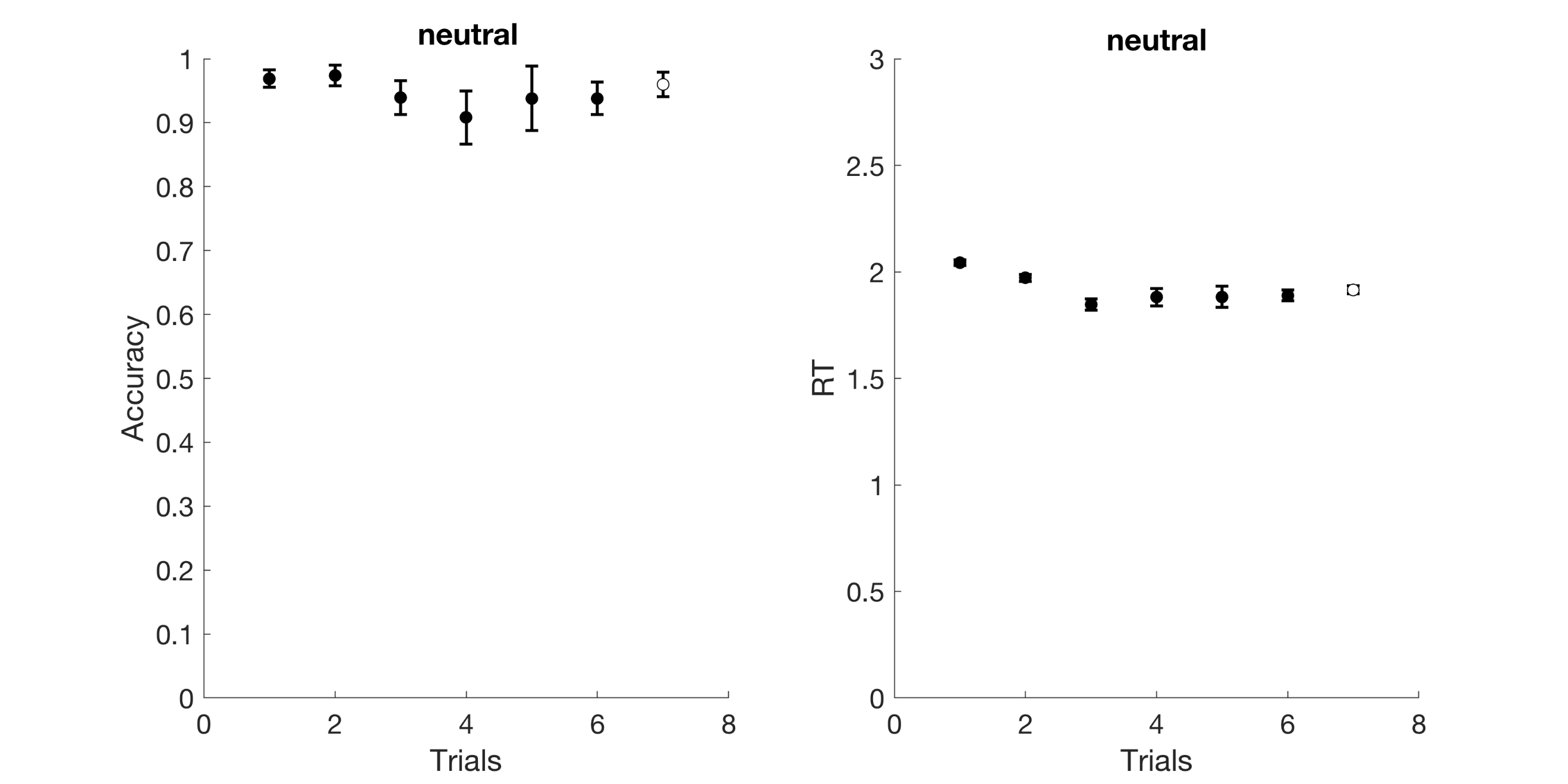
Figure S25. Left panels: Music listening group’s performance in emotion recognition for ‘Neutral’ as a function of number of trials for the average accuracy. Right panels: Music listening group’s performance in emotion recognition for ‘Neutral’ as a function of number of trials for the average reaction time (RT). The error bars represent the standard errors of the means, and the white circle the follow-up measurement of ‘Neutral’ recognition accuracy and RT. Note: neutral had no variations in intensity.


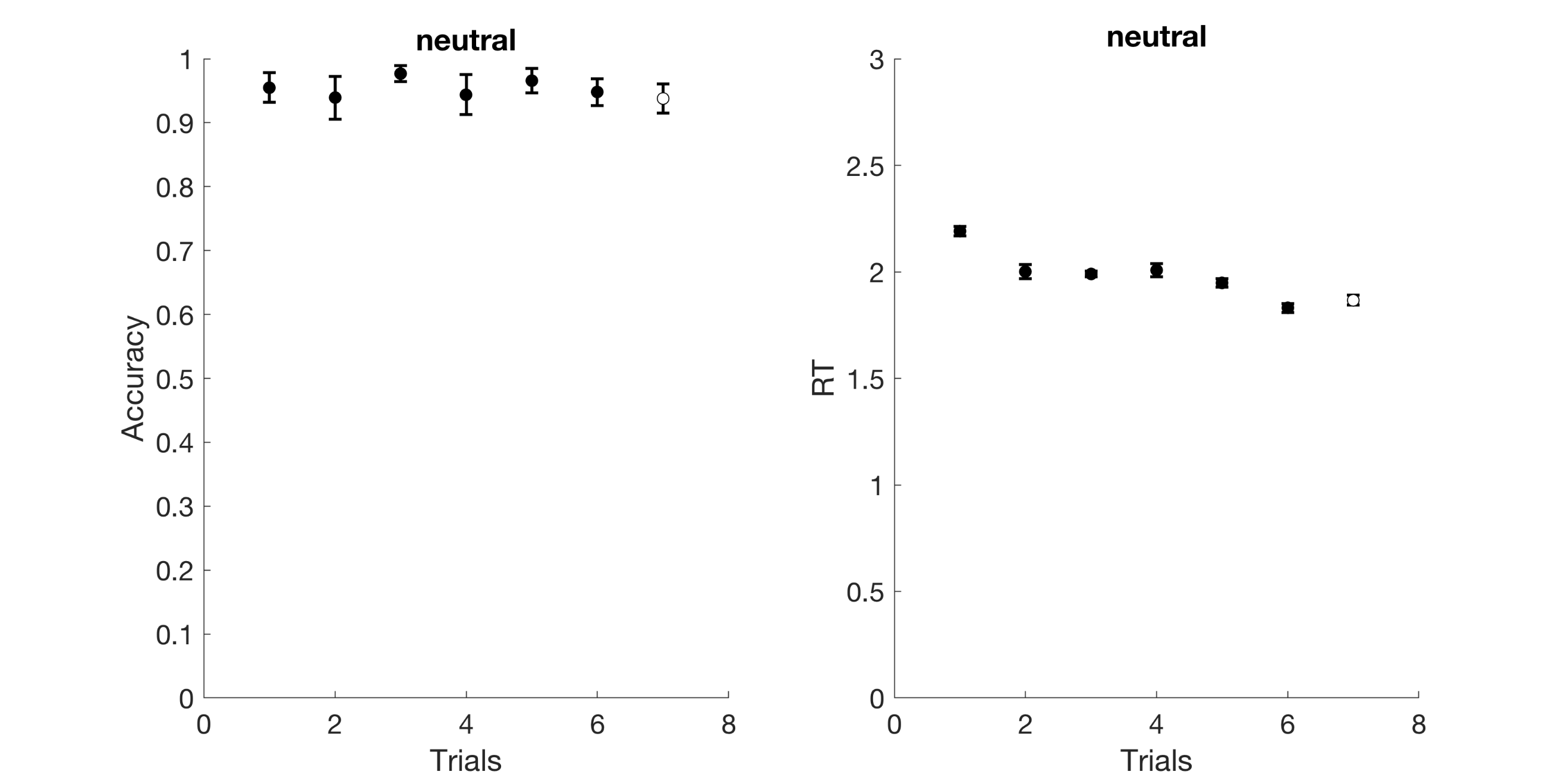
Figure S26. Left panels: Control group’s performance in emotion recognition for ‘Neutral’ as a function of number of trials for the average accuracy. Right panels: Control group’s performance in emotion recognition for ‘Neutral’ as a function of number of trials for the average reaction time (RT). The error bars represent the standard errors of the means, and the white circle the follow-up measurement of ‘Neutral’ recognition accuracy and RT. Note: neutral had no variations in intensity.

**Exploratory analysis**

To examine whether there was a significant correlation between participants’ AQ scores and changes in mood by the end of the study we tested the correlation between positive PANAS difference scores (i.e. the difference between the last and first measure on the positive scale of the PANAS), and between AQ scores and negative PANAS difference scores (i.e. the difference between the last and first measure on the negative scale of the PANAS). Directional (one-tailed) Pearson’s correlation analyses were carried out separately for the three groups (music training, music listening and control), and results showed a significant negative correlation between negative PANAS difference scores and AQ scores in the MT group, *r* = -.623, p = .027 (Figure S26). Hence, individuals that received music training and scored higher on the AQ showed a greater decrease in negative mood.


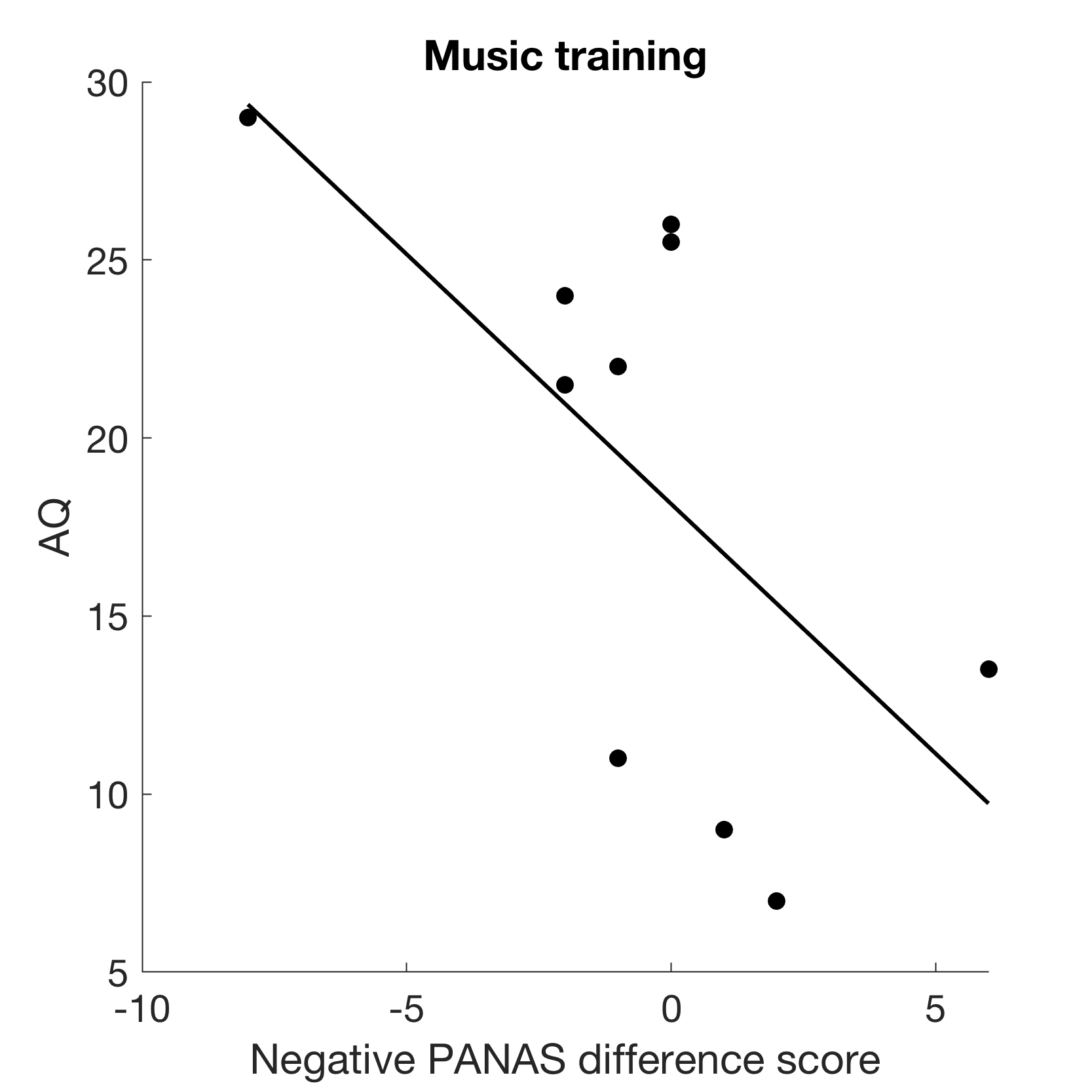


Figure S27. Relation between individuals’ negative PANAS difference scores (calculated for each individual by subtracting the last measure of negative PANAS from the first measure obtained at the beginning of the study) and AQ scores for the music training group.

**Example videos for Supplememtal Files**

*Simultaneity judgement task*

**fb_0 (Converted)**

Clip of flash and beep stimuli with stimulus onset asynchronies (SOA) = 0 (in-synch)

**fb_-133 (Converted)**

Clip of flash and beep stimuli with stimulus onset asynchronies (SOA) = -133 miliseconds (audio-leading, beep appeared first)

**fb_133 (Converted)**

Clip of flash and beep stimuli with stimulus onset asynchronies (SOA) = 133 miliseconds (visual-leading, flash appears first)

**FV_0 (Converted)**

Clip of face and voice stimuli with stimulus onset asynchronies (SOA) = 0 (in-synch)

**FV_-200 (Converted)**

Clip of face and voice stimuli with stimulus onset asynchronies (SOA) = -200 miliseconds (audio-leading, voice appears first)

**FV_200 (Converted)**

Clip of face and voice stimuli with stimulus onset asynchronies (SOA) = 200 miliseconds (visual-leading, face appears first)

*Emotion Recognition task*

**F01-joy-high**

Clip of facial expression stimuli of the emotion ‘joy’ in high intensity

**F01-joy-med**

Clip of facial expression stimuli of the emotion ‘joy’ in medium intensity

**F01-joy-low**

Clip of facial expression stimuli of the emotion ‘joy’ in low intensity
